# Remarkable acceleration of a DNA/RNA inter-strand functionality transfer reaction to modify a cytosine residue: the proximity effect via complexation with a metal cation

**DOI:** 10.1093/nar/gku538

**Published:** 2014-06-21

**Authors:** Daichi Jitsuzaki, Kazumitsu Onizuka, Atsushi Nishimoto, Ikuya Oshiro, Yosuke Taniguchi, Shigeki Sasaki

**Affiliations:** Graduate School of Pharmaceutical Sciences, Kyushu University, 3–1–1 Maidashi, Higashi-ku, Fukuoka 812–8582 Japan, and CREST, Japan Science and Technology Agency, 4–1–8 Motomachi, Kawaguchi, Saitama 332–0012, Japan

## Abstract

Modified nucleosides in natural RNA molecules are essential for their functions. Non-natural nucleoside analogues have been introduced into RNA to manipulate its structure and function. We have recently developed a new strategy for the *in situ* modification of RNA based on the functionality transfer reaction between an oligodeoxynucleotide probe and an RNA substrate. 2′-Deoxy-6-thioguanosine (6-thio-dG) was used as the platform to anchor the transfer group. In this study, a pyridinyl vinyl ketone moiety was newly designed as the transfer group with the expectation that a metal cation would form a chelate complex with the pyridinyl-2-keto group. It was demonstrated that the (*E*)-pyridinyl vinyl keto group was efficiently and specifically transferred to the 4-amino group of the opposing cytosine in RNA in the presence of NiCl_2_ with more than 200-fold accelerated rate compared with the previous system with the use of the diketo transfer group. Detailed mechanistic studies suggested that NiCl_2_ forms a bridging complex between the pyridinyl keto moiety and the N7 of the purine residue neighboring the cytosine residue of the RNA substrate to bring the groups in close proximity.

## INTRODUCTION

Genome science has advanced rapidly over the past two decades, and the remarkable achievements from this period are best exemplified by the discoveries of the important roles of non-coding DNA and RNA. In particular, knowledge of the post-transcriptional chemical modification of non-coding RNA, a widely observed phenomenon, has been considerably broadened, and more than 100 chemical modifications have been determined (1–8). Of particular interest, a chemical modification of mRNA may alter genetic information via ribonucleic acid (RNA) editing by deamination of cytidine to uridine and adenosine to inosine (9,10). 5-Methylcytosine (11,12) and *N*6-methyladenosine (13) have been recently identified in RNA and have attracted attention because of their potential functions in diverse biological processes (14). Endogenous and exogenous chemical entities may also modify RNA and have significant effects, such as inhibition of translation by anti-cancer chemotherapy agents (15) and defective protein synthesis resulting from oxidation with reactive oxygen species (16–18). As advanced sequencing technology has become available, there is an urgent need for chemical tools that are suitable for a variety of investigations on structure and function of RNA. These include site-specific alkylation (19–21), photo-affinity labeling (22,23), site-specific chemical labeling of long RNA molecules (24), chemically modified ribozymes (25) and random acylation of the 2′-hydroxyl group of RNA for profiling (26), among others (27). To attain biological functions through the modification of RNA, it is desirable for a method to be utilized *in situ*.

**Figure 1.**
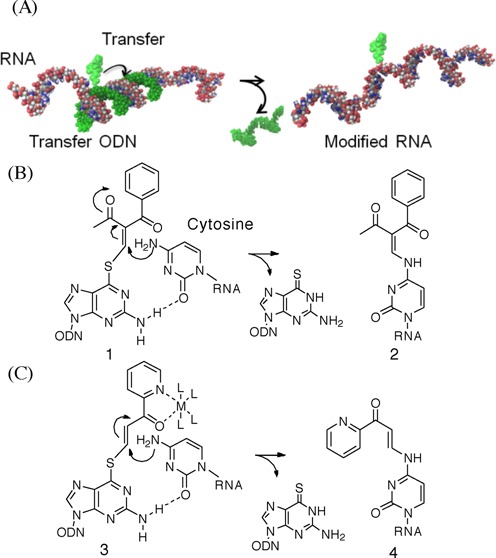
(**A**) Conceptual illustration of the inter-strand functionality transfer from the ODN probe to the RNA substrate within the hybridized complex. (**B**) The 4-NH_2_ group of cytidine in RNA participates in a Michael addition to the vinyl group of the 2-vinylidene-1,3-diketo moiety, and the following elimination of 6-thio-dG accomplishes an *S* to *N* functionality transfer. (**C**) Design of the pyridinyl vinyl keto group as a new transfer group in anticipation of activation through the formation of a metal chelation complex.

Recently, we developed a functionality transfer reaction for the site-specific modification of RNA using an ODN (oligodeoxynucleotide) probe incorporating *S*^6^-functionalized-6-thio-2′-deoxyguanosine (**1**) (Figure 1A). The 2-vinyliden-1,3-diketo moiety was first determined to be a transfer group, and the transfer was accomplished by a sequential reaction of a Michael addition by the 4-amino group of the cytosine base followed by β-elimination of 6-thio-dG (Figure 1B) (28). Subsequently, it was found that the transfer reaction was enhanced with selectively for the 2-amino group of the guanine base at alkaline pH or in the presence of NiCl2 at neutral pH (29,30). This method was applied to the site-specific labeling of RNA (31) and *O*^6^-methyl guanosine-containing DNA (32). The functionality transfer ODN probe (FT-ODN) is advantageous for RNA modification in that the driving force to initiate the transfer reaction is duplex formation with the target RNA. This feature represents a contrast to other methods that use activation stimuli such as photo-irradiation (21) or oxidation (33). However, it was observed that the 2-vinyliden-1,3-diketo moiety is not sufficiently stable or reactive as a transfer group for the modification of the cytosine residue. In this study, to meet the conflicting requirements of stability and reactivity, a pyridinyl vinyl ketone moiety (Pyk) was designed as a new transfer group with the anticipation that activation would occur through complexation with a metal cation, as illustrated by **3**. This activation mechanism resembles the activation of DNA alkylating agents that have a quinolin-8(5H)-one substructure (34–36). This FT-ODN functionalized with an *S*^6^-vinyl pyridinyl ketone moiety showed higher stability in a buffer solution and also demonstrated, in the presence of NiCl_2_, a remarkably efficient transfer reaction with high selectivity for the 4-amino group of the opposing cytidine in RNA with more than 200-fold accelerated rate. A detailed mechanistic study revealed that the transfer reaction occurred specifically with (*E*)-geometry of the thiovinyl moiety and that NiCl_2_ effectively brings the two reactants in close proximity by forming a bridging complex between the FT-ODN and N7 of the purine residue neighboring the cytidine of the target RNA. In this manuscript, we describe in detail the design, synthesis and evaluation of the transfer reaction, as well as a mechanistic study on the effect of metal complexation.

## MATERIALS AND METHODS

Reagents and solvents were purchased from commercial suppliers and were used without purification. ^1^H-, ^13^C-NMR spectra were recorded on Bruker Avance-III. 2D-NMR spectra were measured on Varian Inova 500. IR spectra were recorded on Perkin Elmer Spectrum One FT-IR spectrometer. ESI-HRMS spectra were measured on Applied Biosystems Mariner Biospectrometry Workstation using neurotensin, angiotensin I, bradykinin and picolinic acid as internal standards. MALDI-TOFMS spectra were recorded on BRUKER DALTONICS microflex-KS Linear mass spectrometer.

### Synthesis of the precursors for pyridinyl vinyl keto transfer groups (5–7)

#### 1-(Pyridin-2-yl)prop-2-yn-1-one (5)

A solution of ethynylmagnesium bromide (0.5 M in THF, 20 mL, 10.0 mmol) was added into a solution of 2-cyanopyridine (517 mg, 4.97 mmol) in dry THF (tetrahydrofuran) (3 ml) under argon atmosphere at 0°C. The mixture was warmed to room temperature during 45 min, and again cooled at 0°C. The reaction mixture was quenched with 1% aqueous H_2_SO_4_ and then neutralized with saturated aqueous NaHCO_3_. Precipitates were filtered off through a Celite pad, and the filtrate was extracted with AcOEt. The organic layers were washed with brine, dried over Na_2_SO_4_ and evaporated. The residue was chromatographed on a silica gel column (KANTO 60N, 10 g, Hex-AcOEt = 5:1, v/v) to give **5** as colorless needles (45 mg) in 7% yield. mp: 97–99°C (lit (37) 100–105°C, decomposition). IR (solid) cm^−1^: 3149, 2088, 1653, 1584. ^1^H-NMR (500 MHz, CDCl_3_) δ ppm: 8.76 (1H, ddd, *J* = 4.7, 1.7, 1.0 Hz), 8.07 (1H, ddd, *J* = 7.8, 1.2, 1.0 Hz), 7.95 (1H, ddd, *J* = 7.8, 7.7, 1.7 Hz), 7.63 (1H, ddd, *J* = 7.7, 4.7, 1.2 Hz), 3.97 (1H, s). ^13^C-NMR (125 MHz, CD_3_CN) δ ppm: 179.0, 153.5, 150.8, 138.4, 129.3, 123.8, 84.7, 81.9. ESI-HRMS (*m/z*): calcd for C_8_H_5_NO 132.0444 ([M+H]^+^), found 132.0449.

#### (*E*)-, (Z)-3-Iodo-1-(pyridin-2-yl)prop-2-en-1-one (6 and 7)

A solution of ethynylmagnesium bromide (0.5 M in THF, 38.4 ml, 19.2 mmol) was added into a solution of 2-cyanopyridine (1.0 g, 9.61 mmol) in dry THF (6 mL) under argon atmosphere at 0°C. The mixture was warmed to room temperature during 30 min, and again cooled at 0°C. The reaction mixture was quenched with 10% aqueous HI and stirred for 10 min, then neutralized with saturated aqueous NaHCO_3_. Precipitates were filtered off through a Celite pad and the filtrate was extracted with AcOEt. The organic layers were washed with brine, dried over Na_2_SO_4_ and evaporated. The residue was chromatographed on a silica gel column (KANTO 60 N, 50 g, Hex-AcOEt = 5:1, v/v) to give **6** (780 mg, 38%) and **7** (69 mg, 3%) as a viscous oil.

**(*E*)-6**: IR (film) cm^−1^: 1673, 1559, 1310, 1285, 1229. ^1^H-NMR (500MHz, CD_3_CN) δ (ppm): 8.71 (1H, ddd, *J* = 4.8, 1.7, 1.0 Hz), 8.57 (1H, d, *J* = 15.0 Hz), 8.18 (1H, d, *J* = 15.0 Hz), 8.06 (1H, ddd, *J* = 7.8, 1.2, 1.0 Hz), 7.94 (1H, ddd, *J* = 7.8, 7.6, 1.7 Hz), 7.58 (1H, ddd, *J* = 7.6, 4.8, 1.2 Hz). ^13^C-NMR (125 MHz, CD_3_CN) δ (ppm): 187.6, 153.3, 150.2, 141.3, 138.5 128.6, 123.9, 101.8. ESI-HRMS (*m/z*): calcd for C_8_H_7_INO^+^ [M+H]^+^, 259.9567; found 259.9556.

**(*Z*)-7**: IR (film) cm^−1^: 1678, 1569, 1325, 1266, 1230. ^1^H-NMR (500MHz, CD_3_CN) δ (ppm): 8.70 (1H, ddd, *J* = 4.8, 1.7, 1.0 Hz), 8.62 (1H, d, *J* = 8.8), 8.10 (1H, ddd, *J* = 7.8, 1.0, 1.2 Hz), 7.96 (1H, ddd, *J* = 7.8, 7.6, 1.7 Hz), 7.70 (1H, d, *J* = 8.8 Hz), 7.58 (1H, ddd, *J* = 7.6, 4.8, 1.2 Hz). ^13^C-NMR (125 MHz, CD_3_CN) δ (ppm): 190.0, 154.1, 150.0, 138.5, 133.0, 128.7, 123.6, 95.6. ESI-HRMS (*m/z*): calcd for C_8_H_7_INO^+^ [M+H]^+^, 259.9567; found 259.9556.

#### (*E*)-1-(5-Ethynylpyridin-2-yl)-3-iodoprop-2-en-1-one (8)

A solution of ethynylmagnesium bromide in THF (0.5 M solution, 2.5 ml, 1.25 mmol) was added into a solution of 5-ethynylpicolinonitrile (38) (80 mg, 0.625 mmol) in THF (1.5 mL) at 0°C under an argon atmosphere. After stirring at room temperature for 1 h 10 min, 20% aqueous HI (13 mL) were added to the reaction mixture at 0°C, and then the mixture was stirred for an additional 10 min at the same temperature. Saturated aqueous NaHCO_3_ (20 mL) was added to the reaction mixture and the inorganic precipitate was removed by filtration through a Celite pad. The filtrate was extracted with AcOEt (30 mL×2). The combined organic layers were washed with brine (30 mL), dried over Na_2_SO_4_, filtered and concentrated under reduced pressure to give a crude product, which was purified by flash column chromatography (FUJI SYLISIA FL60D, 15 g, Hex-AcOEt = 20:1, v/v) to give **8** as a yellow foam (17 mg, 0.0608 mmol, 10%). IR (film) cm^−1^: 3238, 3068, 2927, 2108, 1662, 1581, 1559, 1552, 1371, 1316, 1201,1016. ^1^H-NMR (500MHz, CD_3_CN) δ (ppm): 8.79 (1H, dd, *J* = 2.0, 1.0 Hz), 8.53 (1H, d, *J* = 15.0 Hz), 8.21 (1H, d, *J* = 15.0 Hz), 8.04 (1H, dd, *J* = 8.1, 1.0 Hz), 8.01 (1H, dd, *J* = 8.1, 2.0 Hz), 3.80 (1H, s). ^13^C-NMR (125 MHz, CD_3_CN) δ (ppm): 186.8, 152.8, 152.2, 141.5, 141.0, 123.8, 123.4, 102.5, 85.3, 80.6. ESI-HRMS (*m/z*): calcd for C_10_H_7_INO^+^ [M+H]^+^, 283.9567; found 283.9567.

#### Model study of *S*^6^-functionalization of 6-thio-2′-deoxyguanosine (6-thio-dG) and determination of the (*E*)- and (*Z*)-isomer ratio by ^1^H-NMR

Pyridinyl keto derivatives (**5–7**) were reacted with TBS-protected 6-thio-dG (**9**) (37) in MeOH in the presence of TEA at room temperature (Scheme 2). The ratio of the (*E*)- and (*Z*)-isomers of the *S*^6^-vinylated product (**10** or **11**) was estimated by the integration of the vinylic protons in the NMR spectrum (Supplementary Figure S2).

**Figure 2.**
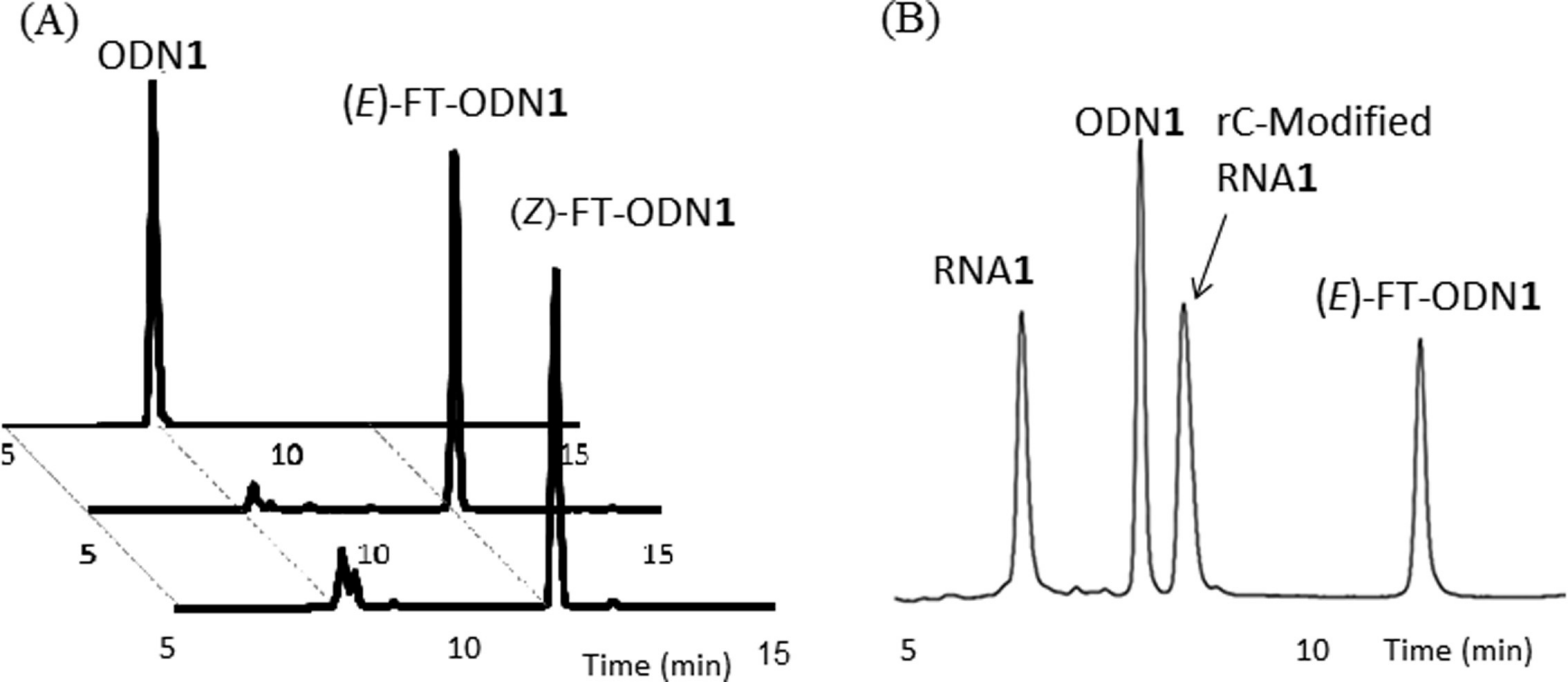
(**A**) Preparation of (*E*)- and (*Z*)-FT-ODN**1**. (**B**) High pressure (or high performance) liquid chromatography (HPLC) trace of the reaction indicating 55% transfer yield of rC-modified RNA**1**.

**Figure 3.**
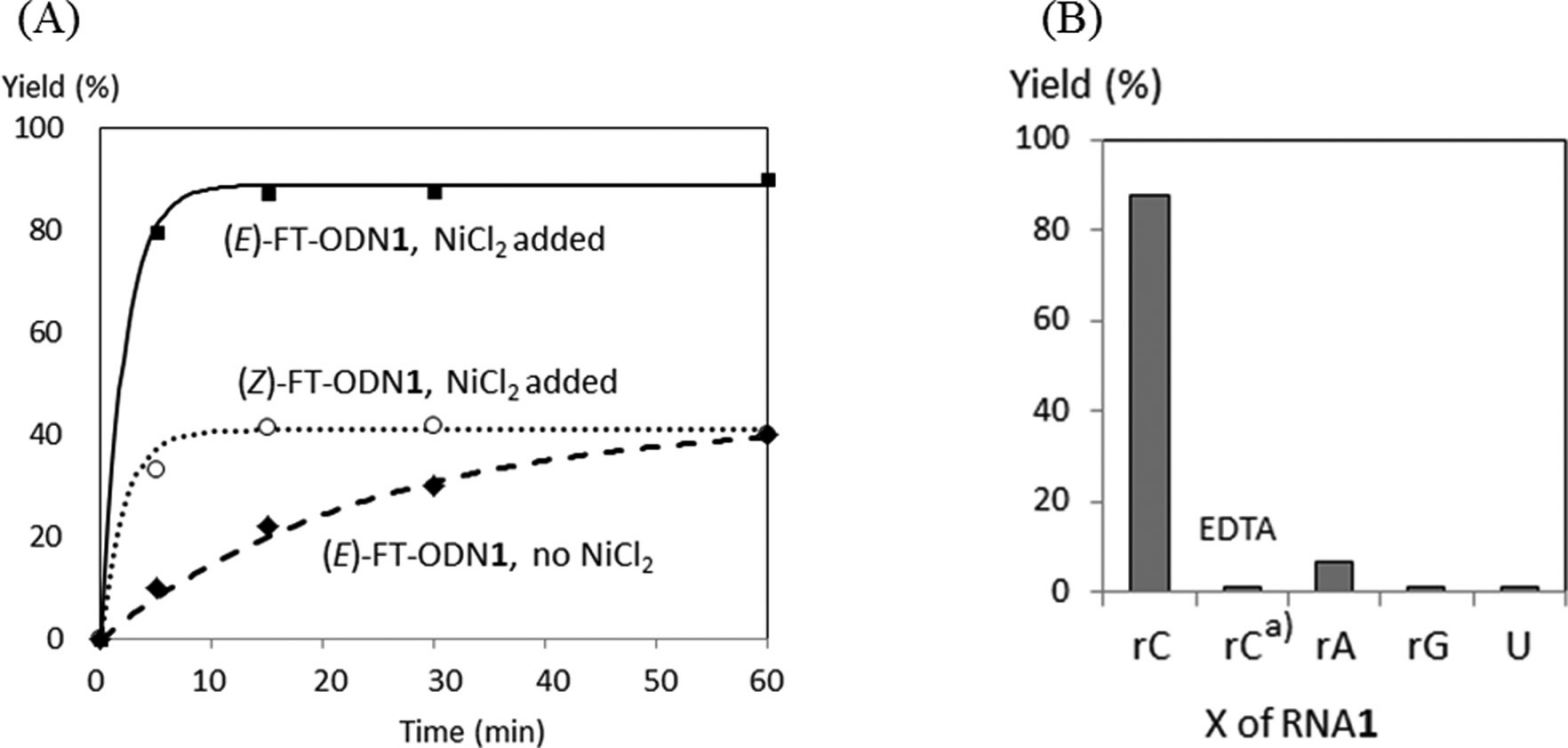
Comparison of the transfer yields for RNA**1**. (**A**) The time course of the transfer yields. Closed squares: (*E*)-FT-ODN**1** in the presence of NiCl_2_; open circles: (*Z*)-FT-ODN**1** in the presence of NiCl_2_; closed diamonds: (*E*)-FT-ODN**1** in the absence of NiCl_2_. (**B**) The transfer yields at 10 min. X represents the nucleotide opposite to the functionalized 6-thio-dG of FT-ODN**1**. ^a^1 mM of Ethylenediaminetetraacetic acid (EDTA) was added. The reaction was performed at 37°C using 5 μM of RNA**1** and 7.5 μM of FT-ODN**1** in a buffer containing 50 mM HEPES (2-(4-(2-hydroxyethyl)piperazin-1-yl)ethane-1-sulfonic acid) and 100 mM NaCl at pH 7 in the presence or absence of NiCl_2_ (5 μM).

**Figure 4.**
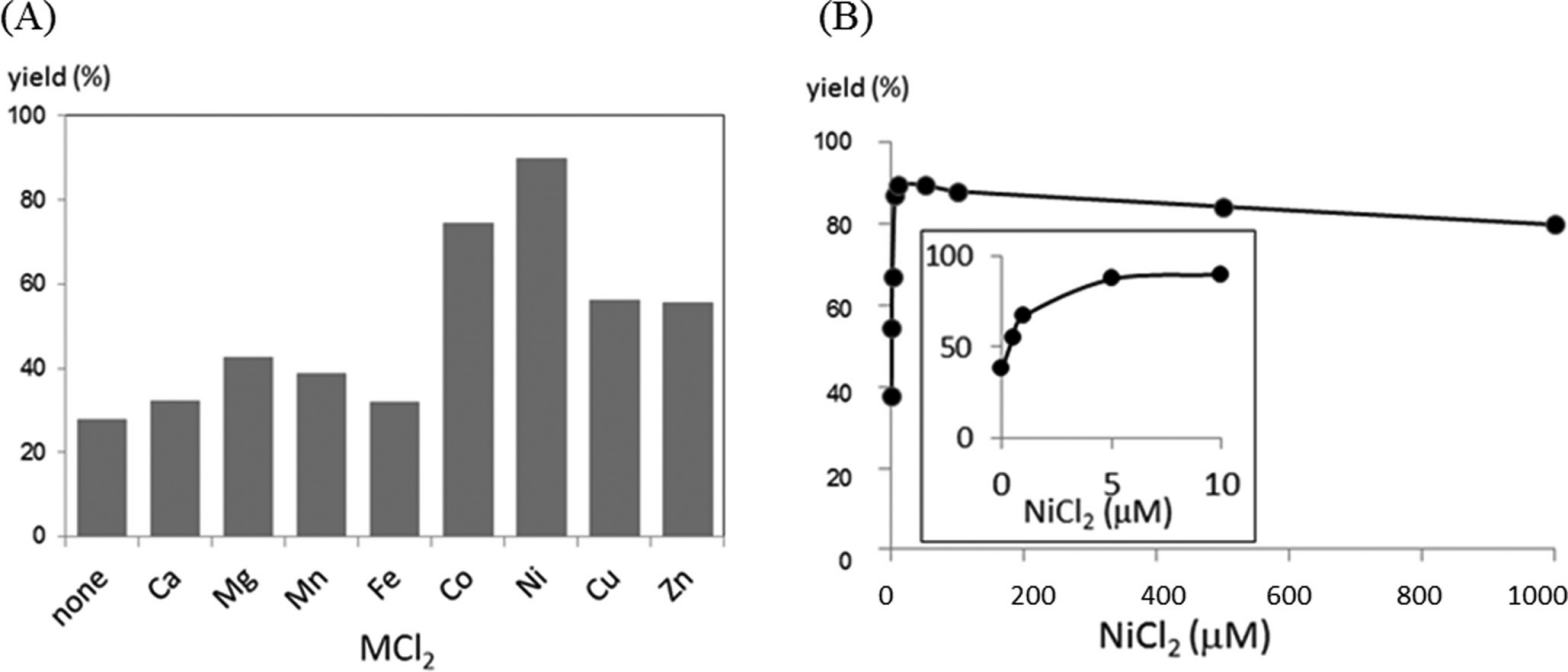
Effect of metal cations on the transfer yield. (**A**) Comparison of the yields in the presence of 5 μM of MCl_2_. (**B**) Effect of the concentration of NiCl_2_. The transfer yields at 1 h are compared. The reaction conditions are the same as described in the footnote to Figure 3 except that the yield was measured after 60 min.

**Figure 5.**
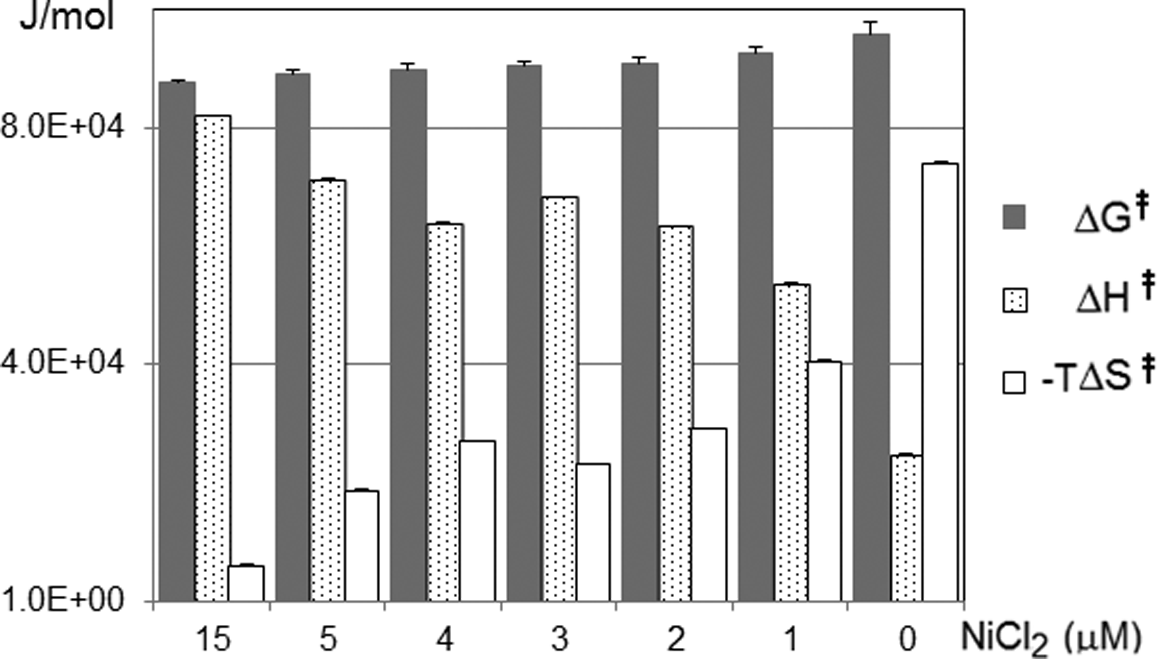
Kinetic parameters at different NiCl_2_ concentrations. The error bars represent the standard deviation from the mean of the data obtained at different temperature. Experimental details and data are described in Supporting Information.

**Figure 6.**
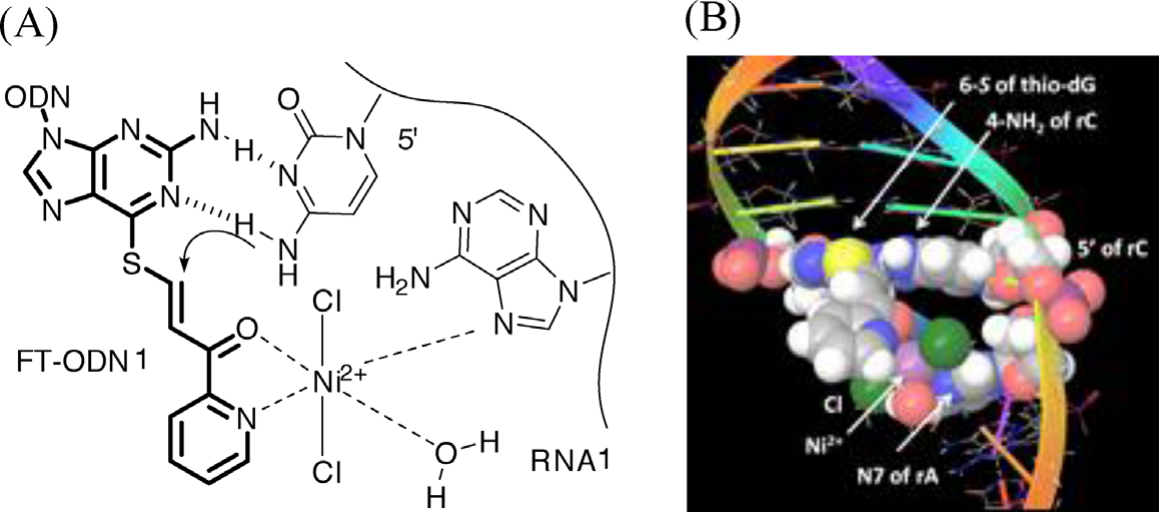
(**A**) A hypothetical complex of Ni^2+^ with the pyridinylketo unit and N7 of an adenine residue at the 5′ side. (**B**) Molecular modeling of the complex in the ODN**1**/RNA**1** duplex.

**Figure 7.**
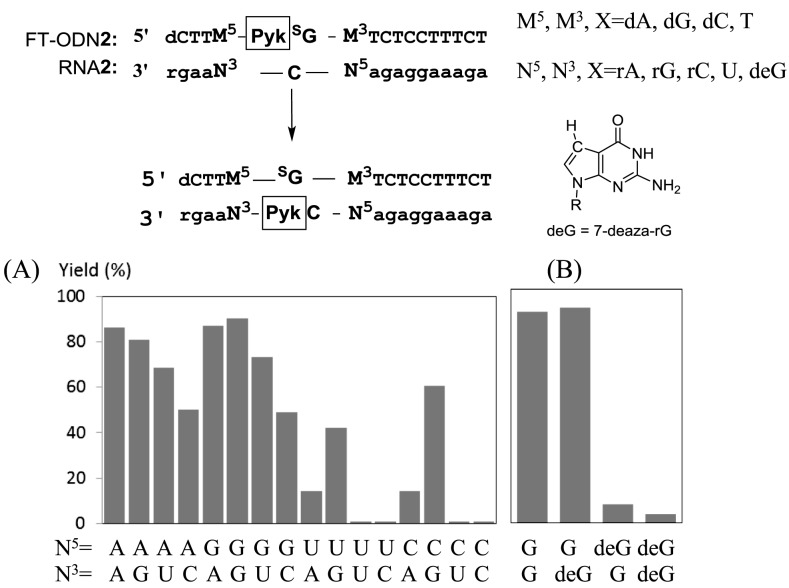
(**A**) Effect of flanking base pairs on the transfer yield. M^5^ and M^3^ of FT-ODN**2** are complementary bases corresponding to N^3^ and N^5^ of RNA**2**, respectively. (**B**) 7-Deaza-rG (deG) was present in RNA**2.** The reaction conditions are the same as described in the footnote to Figure 3 except that the yield was measured after 60 min.

**Scheme 1.**
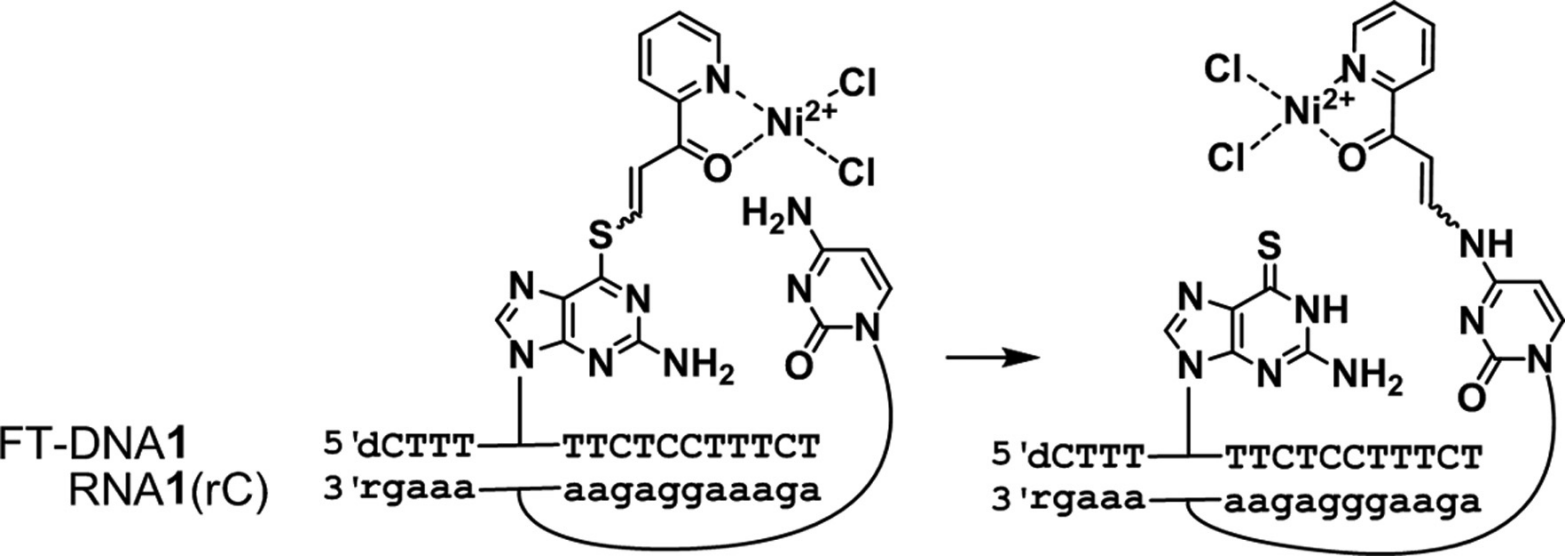
The functionality transfer reaction from FT-ODN**1** to RNA**1** (rC).

**Scheme 2.**
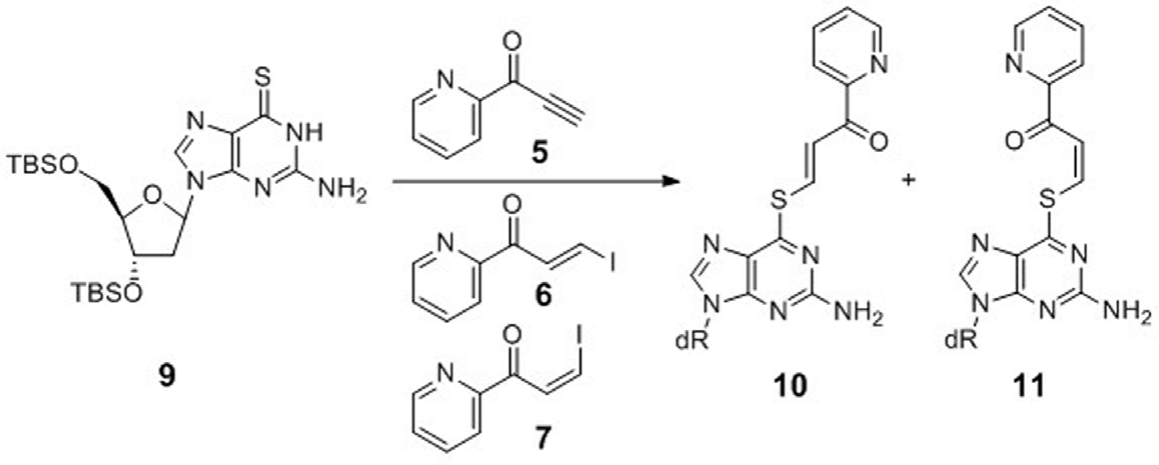
Model experiments to determine (*E*)- and (*Z*)-stereochemistry of the transfer unit of 6-thio-dG.

**Scheme 3.**
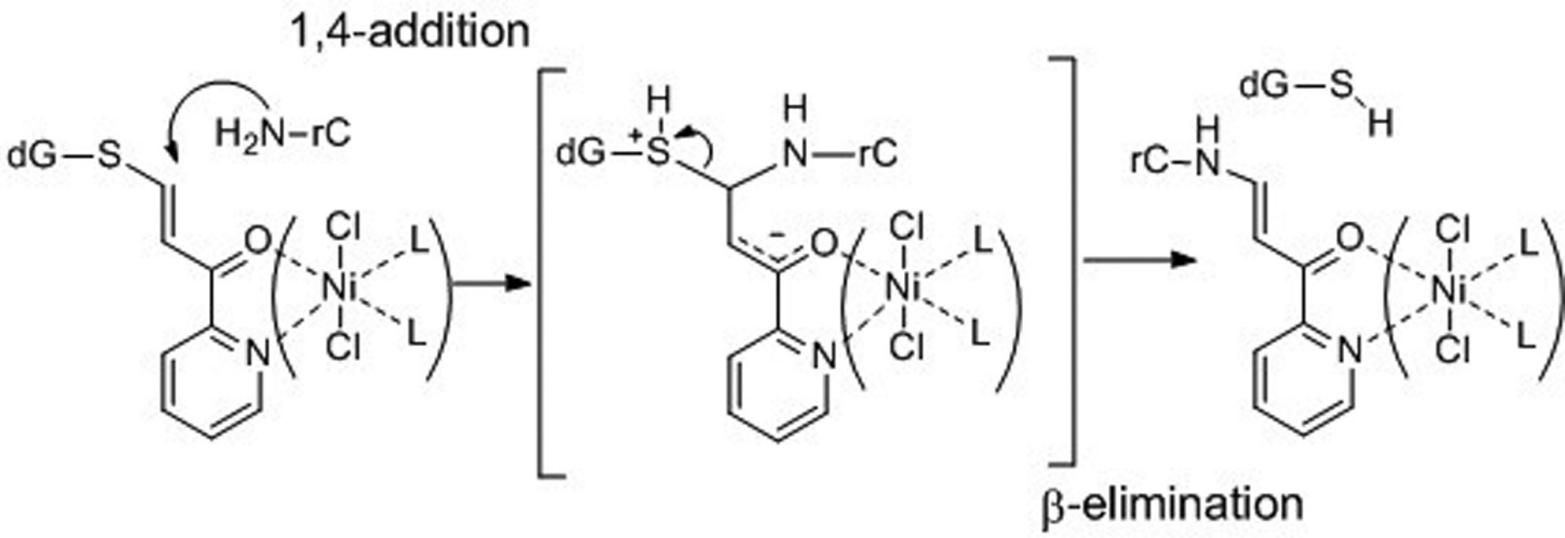
1,4-Michael addition and following β-elimination leading to *S*-to-*N* transfer reaction.

**Chart 1.**
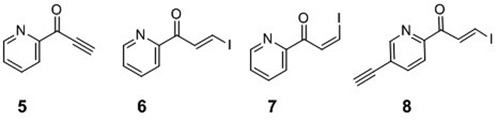
The precursors used for attaching pyridinyl keto transfer groups to 6-thio-2′-deoxyguanosine in ODN probe.

#### A general procedure for preparation of the functionality transfer (FT-ODN)

The ODNs incorporating 6-thio-2′-deoxy-guanosine (ODN**1** and **2**) were synthesized as previously reported (39). A solution of ODN**1** (50 μM) and the alkylating agent (**6**, 500 μM) in 25 mM carbonate buffer was incubated at pH 10 and 0°C for 30 min, and analyzed by HPLC using the following conditions. Column: SHISEIDO C18, 4.6 × 250 mm, solvents, A: 0.1M TEAA, B: CH_3_CN, B 10% to 30% /20 min, 30% to 100% /25 min, linear gradient; flow rate at 1.0 ml/min, UV monitored at 254 nm. Examples of HPLC changes are shown in Figure 2. The MALDI-TOFMS data of the prepared ODN are summarized in Supplementary Table S1. FT-ODN was used without purification for the transfer reaction.

**Chart 2.**
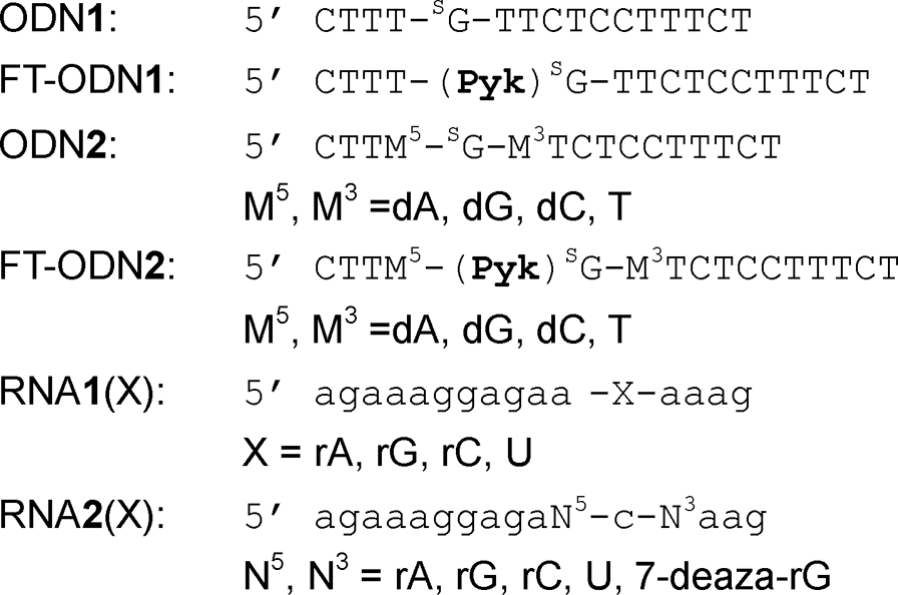
Sequences of ODNs and RNAs used in this study.

#### The functionality transfer reaction and determination of the structure of its product

The above mixture employed for the preparation of FT-ODNs was used without purification for the transfer reaction. The mixture was diluted with water, and a buffer solution was prepared such that the reaction mixture contained 5 μM of RNA (1 or 2), 7.5 μM of FT-ODN (1 or 2), 50 mM HEPES and 100 mM NaCl at pH 7. The concentration of NiCl_2_ was adjusted according to the requirements of the reactions. The reaction progress was followed by HPLC, examples of which are shown in Figure 2B. The transfer yield was obtained by quantification of the peaks corresponding to product and RNA**1** or **2**. The rC-modified RNA**1** was analyzed by MS/MS to confirm that the target rC was modified by the transfer reaction (Supplementary Figure S4). To determine the structure of the modified cytidine, the corresponding DNA substrate was used. The modified DNA substrate was purified, reduced with NaBH_4_, HPLC purification and hydrolyzed with bacterial alkaline phosphatase (BAP), nuclease P1 and venom phosphodiesterase (VPDE). The lysates were analyzed by HPLC (Supplementary Figure S5). The authentic sample was synthesized separately (Supplementary Scheme S3) and used for confirmation by HPLC co-injection.

#### Kinetic analysis of the functionality transfer reaction using RNA1 (rC) and (*E*)-FT-ODN1

The reaction was performed at 15, 20, 25, 30 and 35°C with different concentrations of NiCl_2_ (0, 1, 2, 3, 4, 5, 15 μM), and the modified RNA was quantitatively analyzed by HPLC. The reaction was analyzed as a first-order reaction using an initial duplex concentration of 4.5 μM as the reactive duplex formed with (*E*)-FT-ODN**1**, and the remaining concentration (0.5 μM) as the nonreactive duplex formed with (*Z*)-FT-ODN**1**. The activation energy (*E*_a_) was obtained by Arrhenius plots, and kinetic parameters were obtained from the Eyring equation.

#### Molecular modeling of the complex of *S*^6^-functionalized ODN, target RNA and NiCl_2_

The OPLS 2005 parameters installed in MacroModel were modified to calculate the NiCl_2_ complex in water using the model complex structure optimized by ub3lyp/6–31G(d). An octahedral NiCl_2_ complex was assumed to coordinate with the nitrogen and oxygen atoms of the pyridiyl keto transfer moiety, the N7 atom of adenine or guanine and one water molecule. During optimization, the NiCl_2_ complex structure was constrained by setting bond lengths within a range of values. Therefore, the simulated structures illustrate only candidate complexes leading to the transfer reaction.

## RESULTS AND DISCUSSION

### Selective synthesis of the 6-thio-dG monomer functionalized with the (*E*)- or (*Z*)-pyridinyl vinyl keto transfer moiety

Pyridinyl keto transfer groups (Pyk, **5–8**) were used to modify the ODN probe incorporating 6-thio-2′-deoxyguanosine. Ethynyl derivative **5** was synthesized using 2-cyanopyridine and ethynyl magnesium bromide. A mixture of (*E*)- and (*Z*)-iodovinyl derivatives (**6–7**) were obtained by an analogous reaction, except that the reaction mixture was treated with aqueous HI solution and each isomer was separated by column chromatography. The 5-ethynylpyridine derivative **8** was synthesized in a similar fashion from 2-cyano-5-ethynylpyridine, which was prepared from the corresponding 5-bromo derivative. In a preliminary study, ODN**1** containing 6-thio-dG was functionalized using the ethynyl keto pyridine derivative (**5**) to prepare FT-ODN**1**, and it was tested for the transfer reaction with RNA**1** (Scheme 1). The reaction proceeded rapidly, but the formation of the modified RNA reached a plateau of ∼30% yield within 1 h (Supplementary Figure S1). The (*E*)- and (*Z*)-stereochemistry of the transfer unit was suspected to influence reactivity; however, determination of the *E*/Z ratio of the synthesized FT-ODN**1** was not possible by HPLC or gel electrophoresis. The ^1^H-NMR signals of the vinyl keto pyridine moiety are in the same region of nucleobases. Therefore, the *E*/Z ratio was measured in a model reaction using a 6-thio-dG nucleoside in organic solvents (Scheme 2). (*E*)- and (*Z*)-iodovinyl derivatives (**6** and **7**, respectively,) were purified **5** and then reacted with TBS-protected 6-thio-dG (**9**) in methanol in the presence of triethylamine (Scheme 2). The (*E*)- and (*Z*)-ratio (**10** / **11** ratio) was determined by ^1^H-NMR (Supplementary Figure S2). The ethynylketopyridine derivative (**5**) that was used to prepare FT-ODN**1** in a preliminary study produced **10** and **11** in a 56:44 ratio. The pure (*E*)-isomer (**6**) gave **10** and **11** in a 92:8 ratio with selectivity for the (*E*)-isomer; however, the reaction with the pure (*Z*)-isomer (**7**) resulted in only a 35:65 ratio in favor of the (*Z*)-isomer.

### The functionality transfer reaction

ODN**1** containing 6-thio-dG was functionalized with **6** to produce (*E*)-FT-ODN**1** as the sole product (Figure 2A). Assuming that the modification of 6-thio-dG in DNA in aqueous solution occurs in a similar fashion as in organic solvents, FT-ODN**1** was thought to be prepared in a ratio of ∼92:8 in favor of the (*E*)-isomer and is thus identified as (*E*)-FT-ODN**1**. Alternatively, FT-ODN**1** was assumed to form in a 35:65 ratio in favor of the (*Z*)-isomer after reaction with **7** and is represented as (*Z*)-FT-ODN**1**. These isomers were used without separation for the transfer reaction.

The transfer reaction shown in Scheme 1 was performed at 37°C using 5 μM of RNA**1** and 7.5 μM of FT-ODN**1** in 50 mM HEPES buffer containing 100 mM NaCl at pH 7, and progress was analyzed by HPLC. Figure 2B shows an example of an HPLC trace revealing a 55% transfer yield for the reaction between RNA**1**(rC) and (*E*)-FT-ODN**1**. The yields of modified RNA**1** obtained under different conditions are plotted against time in Figure 3A. In the presence of 5 μM NiCl_2_, the transfer reaction with (*E*)-FT-ODN**1** proceeded rapidly and was completed before 10 min to produce modified RNA**2** in ∼90% yield (solid line in Figure 3A). The transfer yield at 10 min was improved from ca. 1% to 90% compared with the previous system with the use of the diketo transfer group,^28^ meaning more than 200-fold rate acceleration. Remarkably, the reaction yield with (*Z*)-FT-ODN**1** only reached a plateau of ∼40% yield in contrast (dotted line in Figure 3A). Assuming that the (*E*)-isomer represents ∼92% of (*E*)-FT-ODN**1** and 35% of (*Z*)-FT-ODN**1**, these results can be interpreted as a result of the high reactivity of the (*E*)-isomer and the lack of isomerization from the *Z*-isomer to the *E*-isomer during the transfer reaction. It should also be noted that addition of NiCl_2_ significantly increased the reaction rate for (*E*)-FT-ODN**1** (continuous line versus dashed line in Figure 3A). Figure 3B summarizes the transfer yields with (*E*)-FT-ODN**1** at 10 min. EDTA completely inhibited the reaction, most likely by trapping metal cations in the buffer, suggesting that metal cations in the buffer also contribute to activation to some extent. A high selectivity for rC was clearly shown in Figure 3B. The site-specific labeling of rC in RNA has been demonstrated by using an acetylene-functionalized pyridinyl keto unit (**8**) for a Cu-catalyzed click reaction with azido derivatives (Biotin-N_3_ and FAM-N_3_) (31)(Supplementary Scheme S2 and Supplementary Figure S3).

A comparison of the effect of other metal cations on activation is displayed in Figure 4A, showing an order of Ni^2+^ > Co^2+^ > Cu^2+^, Zn^2+^ > Ca^2+^, Mg^2+^, Mn^2+^ and Fe^2+^. The rate enhancement by NiCl_2_ was dependent on the concentration, and a maximum was reached at a concentration equimolar to the RNA substrate (Figure 4B inset). Thus, the initial design in which the pyridinyl vinyl ketone unit is activated by forming a chelate complex with NiCl_2_ appeared to be validated by these results. In contradiction to the observed activation by NiCl_2_, the degradation half-life of (*E*)-FT-ODN**1** in the buffer (10–12 h) was not affected by NiCl_2_; i.e. the intrinsic reactivity of (*E*)-FT-ODN**1** was not increased by NiCl_2_. The results posed two questions: the difference in reactivity of the (*E*)-or (*Z*)-isomers in the transfer group, and the mechanism for activation by NiCl_2_.

### Mechanistic study of the activation of the transfer reaction by NiCl_2_

As part of a mechanistic study, the modification site was determined to be the cytosine residue by UPLC/MS/MS (Supplementary Figures S4). The *N*4-alkylation of rC was confirmed by HPLC analysis of the hydrolysates of the modified product using the DNA substrate corresponding to RNA**1** (Supplementary Figures S5 and S6). To obtain further insight into the effect of NiCl_2_ on kinetics, the reaction was performed at different temperatures in the presence of different concentrations of NiCl_2_. As the transfer reaction occurs through an intra-complex interaction, the rate constants were obtained by analyzing the reaction as first-order. The *E*_a_ values were obtained by Arrhenius plots, and kinetic parameters were calculated from the Eyring equation, and are expressed in a bar graph (Figure 5).

The Δ*G*^‡^ value decreased with increasing NiCl_2_ concentration in accord with the fact that NiCl_2_ increased the transfer yield (Figure 4B). The entropy of activation (−TΔ*S*^‡^) was a major component of the Δ*G*^‡^ value in the absence of NiCl_2_ (NiCl_2_, 0 μM). Interestingly, although the transfer reaction was enhanced by NiCl_2_, the enthalpy of activation (Δ*H*^‡^) conversely increased (see also Supplementary Figures S7 and Table S2). The −TΔ*S*^‡^ value decreased significantly in the presence of NiCl_2_, with an accompanying increase of the Δ*H*^‡^ value. At a high concentration of NiCl_2_, the −TΔ*S*^‡^ value was substantially diminished, and the Δ*H*^‡^ value became a major component of the ΔG^‡^ value. In spite of increases in the Δ*H*^‡^ value, larger decreases of the −TΔ*S*^‡^ value reduced the Δ*G*^‡^ value, favorably affecting the reaction. These kinetic data accord with the conflicting properties of (*E*)-FT-DON**1** in that NiCl_2_ increased the reaction rate of the transfer moiety without affecting the intrinsic reactivity of the transfer moiety in the buffer. Considering that the functionality transfer reaction proceeds through a Michael addition of the 4-NH_2_ group and subsequent β-elimination of the 6-thio-dG residue (Scheme 3), a large −TΔ*S*^‡^ value for the reaction in the absence of NiCl_2_ suggests that the 1,4-addition step is most likely a rate-determining step. On the other hand, the small −TΔ*S*^‡^ value and large Δ*H*^‡^ value for the reaction at high NiCl_2_ concentrations may be an indication that the bond-cleaving β-elimination is a rate-determining step.

To satisfy these interpretations, we noted that N7 of the purine residue is a binding site for NiCl_2_ in addition to the pyridinyl keto unit (40). NiCl_2_ forms a particularly stable complex with the N7 of guanine in an extra-helical or terminal position (41). Thus, it was supposed that Ni^2+^ forms complexes with both the pyridinyl ketone of (*E*)-FT-ODN**1** and N7 of the purine residue, either at the 5′ or 3′ side of the opposing rC of the RNA**1** substrate. Such a complex may form an inter-strand bridge between (*E*)-FT-ONN**1** and RNA**1** and bring the 4-NH_2_ group of rC in RNA**1** in proximity with the thio-vinyl reactive site as shown in Supplementary Figure 6A and 6B. Molecular modeling also suggested that such a Ni^2+^-bridged complex formation with (*Z*)-FT-ODN**1** requires distortion of the duplex structure (Supplementary Figure S8). Thus, by predicting a bridging complex with NiCl_2_ as shown in Figure 6, it may be reasonably explained why only the (*E*)-transfer group is highly reactive and how NiCl_2_ activates the transfer reaction without decreasing the stability of FT-ODN. To confirm a significant role of the purine residue, we next investigated effects of base pairs flanking the target rC. The results are summarized in Figure 7A. Flanking bases composed of rA and rG at both 5′ and 3′ sides were the most effective. Efficiency was retained with rG at the 3′ side (N^5^-N^3^ = UG and CG) or rA at the 3′ side (N^5^-N^3^ = UA and CA). In contrast, the transfer reaction remarkably did not occur when the flanking bases were composed of U and rC at both 5′ and 3′ sides (N^5^-N^3^ = UU, UC, CU, CC). 7-Deaza-rG (deG) was used as a control residue, as it lacks an N7 binding site for NiCl_2_. Replacement of rG with deG diminished the transfer reaction (Figure 7B), clearly underscoring the importance of N7. These results have strongly suggested that the formation of a bridging complex with NiCl_2_, N7 of the purine residue and the (*E*)-pyridinyl keto unit as shown in Figure 6 is responsible for activation by NiCl_2._

In conclusion, the highly efficient and site-specific modification of rC in RNA has been successfully demonstrated by a functionality transfer reaction using the (*E*)-pyridinyl vinyl keto transfer group. A remarkable acceleration in the transfer reaction is achieved by the enforced proximity effect mediated by a bridged complex formed between NiCl_2_, the (*E*)-pyridinyl vinyl keto moiety and N7 of purine residues flanking the opposing rC. The transfer rate of the (*E*)-pyridinyl vinyl keto moiety has exhibited more than 200-fold increased reaction rate compared with the diketo transfer group. Notably, NiCl_2_ enhances the transfer reaction without increasing the intrinsic reactivity of the transfer group that is ascribable to the instability of the ODN probe, and a variety of organic functional groups can be easily attached on the specific site of RNA under mild conditions. With regard to its application, it is interesting to investigate the effect on deamination activity and template effect by the specific modification of a single cytidine such as in the large apolipoprotein B mRNA.^9^ The search of compounds that may have an impact on the structure and function of RNA is now ongoing.

## SUPPLEMENTARY DATA

Supplementary Data are available at NAR online.

SUPPLEMENTARY DATA
