# Supplementary material for: Remarkable acceleration of a DNA/RNA inter-strand functionality transfer reaction to modify a cytosine residue: the proximity effect via complexation with a metal cation
Source: Nucleic Acids Res. 2014 Jun 21;42(13):8808–15. doi: 10.1093/nar/gku538 (PMC4117767; doi:10.1093/nar/gku538)
Supplement: SUPPLEMENTARY DATA [file supp_42_13_8808__index.html]

Remarkable acceleration of a DNA/RNA inter-strand functionality transfer reaction to modify a cytosine residue: the proximity effect via complexation with a metal cation — SUPPLEMENTARY DATA 

# Remarkable acceleration of a DNA/RNA inter-strand functionality transfer reaction to modify a cytosine residue: the proximity effect via complexation with a metal cation

## SUPPLEMENTARY DATA

**Files in this Data Supplement:**

- SUPPLEMENTARY DATA
