# Supplementary material for: Remarkable acceleration of a DNA/RNA inter-strand functionality transfer reaction to modify a cytosine residue: the proximity effect via complexation with a metal cation
Source: Nucleic Acids Res. 2014 Jun 21;42(13):8808–15. doi: 10.1093/nar/gku538 (PMC4117767; doi:10.1093/nar/gku538)

Supporting Information

Remarkable Acceleration of a DNA/RNA Inter-Strand Functionality-Transfer Reaction to Modify a Cytosine Residue: the Proximity Effect via Complexation with a Metal Cation

Daichi Jitsuzaki, Kazumitsu Onizuka, Atsushi Nishimoto, Ikuya Oshiro, Yosuke Taniguchi, Shigeki Sasaki*

Graduate School of Pharmaceutical Sciences, Kyushu University, 3-1-1 Maidashi, Higashi-ku, Fukuoka 812-8582 Japan, and CREST, Japan Science and Technology Agency, 4-1-8 Motomachi, Kawaguchi, Saitama 332-0012, Japan.

Scheme S1-S3 S2
Figure S1 and S2 S3
Figure S3 and S4 S4
Figure S5 and S6 S5
Figure S7 and S8 S6
Experimental S7
Table S1. MALDI-TOF/MS data S11
Table S2. S13
References S13
^{13}C- and ^{1}H-NMR spectra S14-S31

Schemes S1-S3

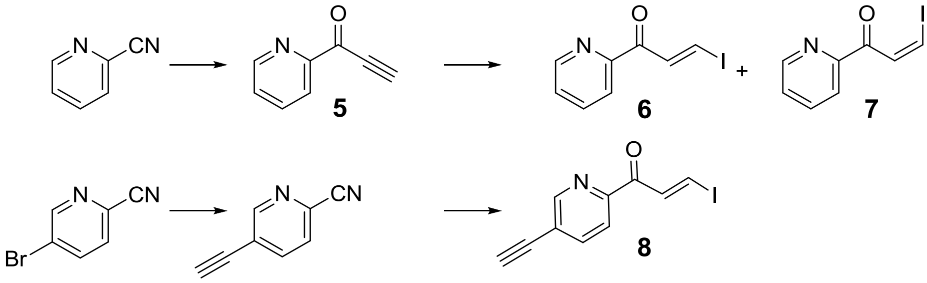

Scheme S1. Synthesis of the (*E*)- and (*Z*)-pyridinyl vinyl keto transfer group.

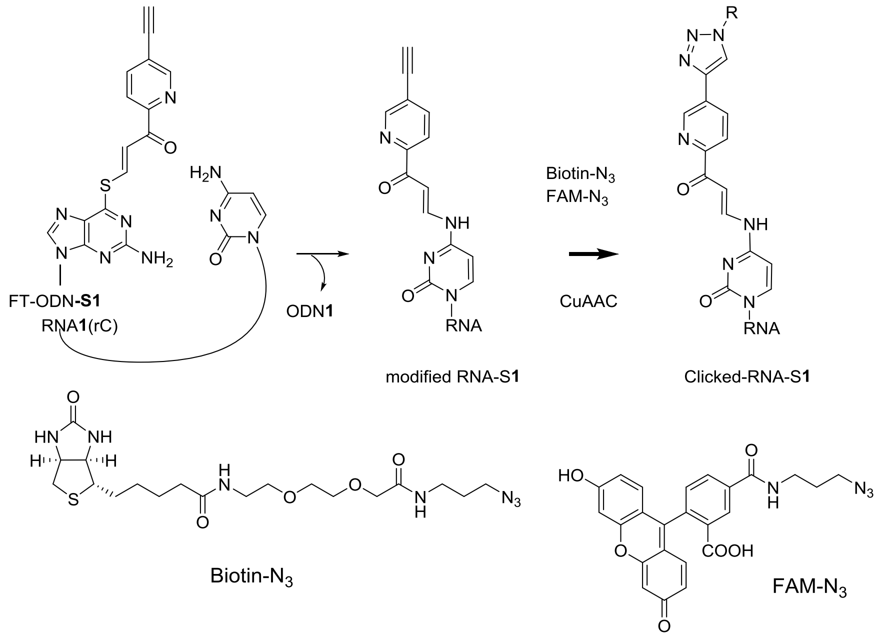

Scheme S2. The functionality transfer reaction using FT-ODN-S**1** and RNA**1**, and the click reaction with biotin-N_3 or FAM-N_3.

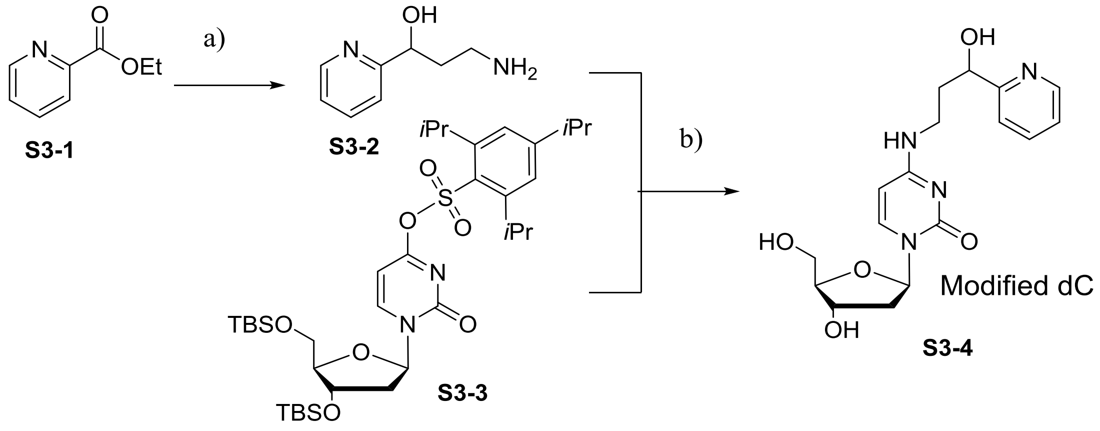

Scheme S3. Synthesis of the authentic sample of the modified-dC.

a) (1) NaH, CH_3CN, toluene, 64 %, (2) $LiAlH_4$, THF, 95%, b) (1) MeOH, 19%, (2) Bu_4NF, THF, 95%

Figures S1-S8

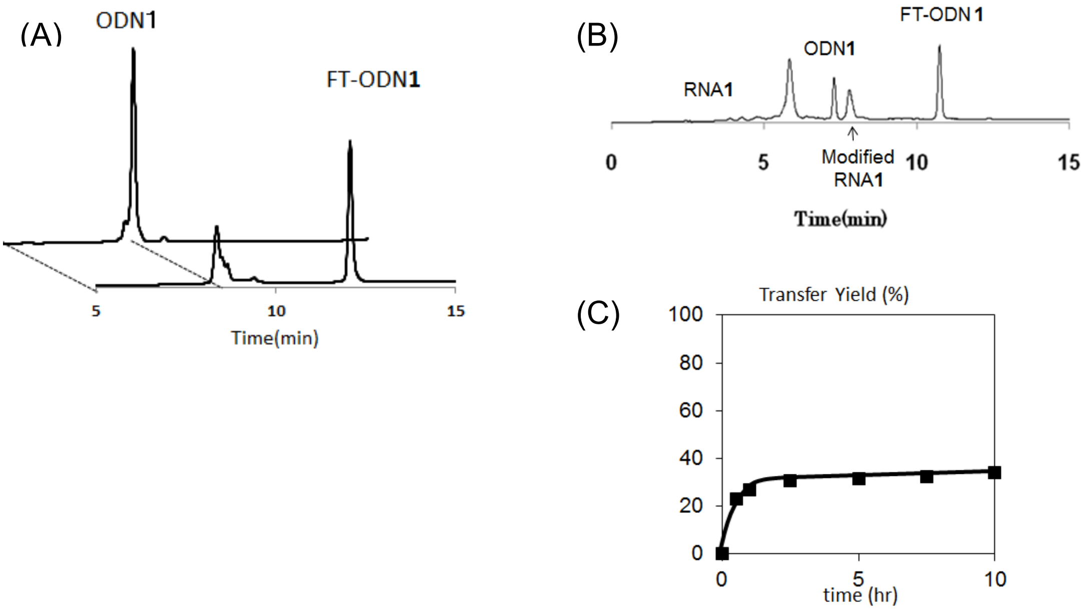

Figure S1. (A) Synthesis of FT-ODN**1** using **5**. ODN**1** was functionalized using 100 μM of ODN and 500 μM of the alkylating agent **5** in 25 mM carbonate buffer at pH 10 and r.t. for 10 min, and analyzed by HPLC. (B) HPLC of the transfer reaction after 2.5 hr using each of 5 μM RNA**1**, 6 μM FT-ODN**1** and 0.6 μM $NiCl_2$. (C) Time course of the transfer reaction using 5 μM RNA**1**, 7.5 μM FT-ODN**1** and 0.75 μM $NiCl_2$.

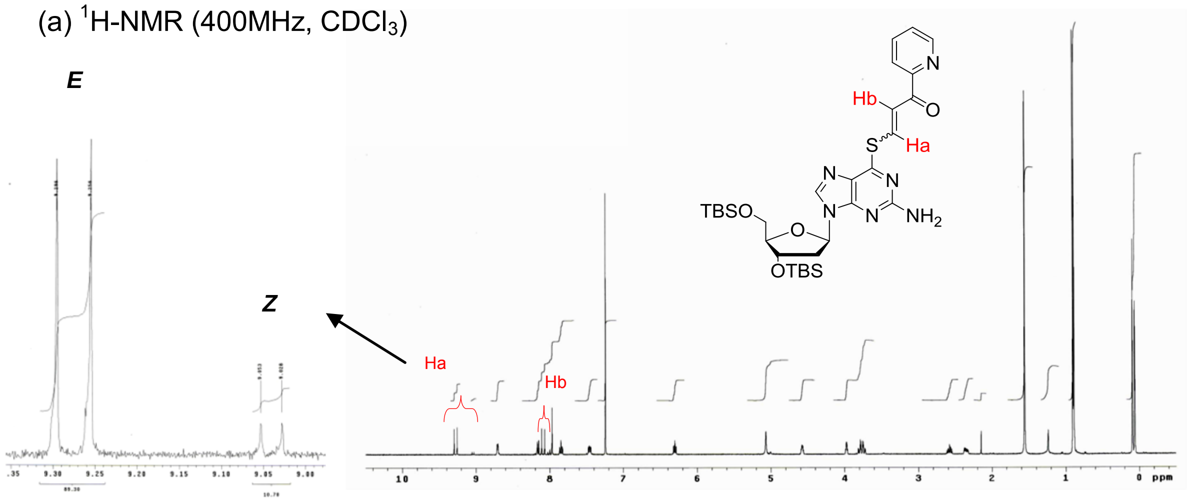

Figure S2. Determination of *E*- to *Z*-ratio by ^{1}H-NMR.

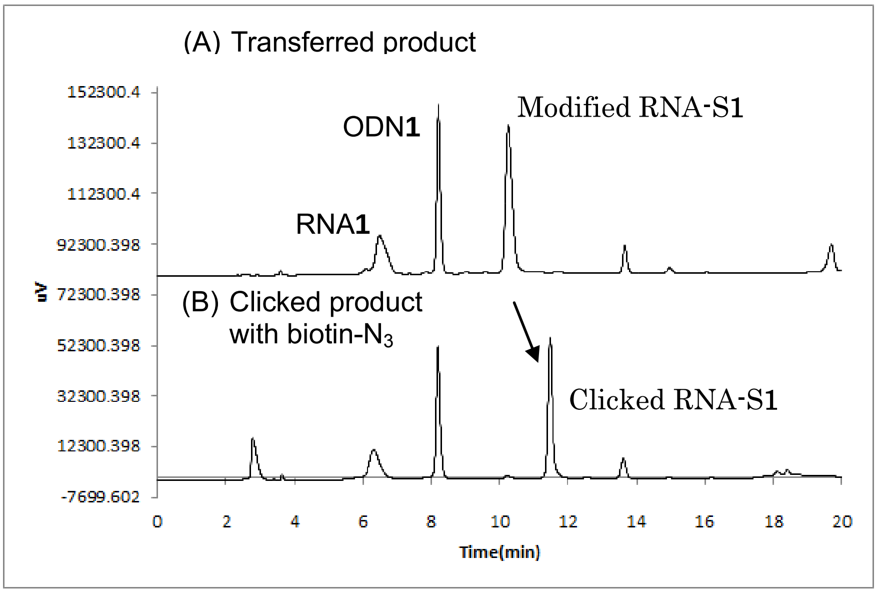

Figure S3. (A) HPLC chart of the mixture after the functionality transfer reaction using FT-ODN-S1 and RNA1. (B) HPLC chart of the mixture of the click reaction.

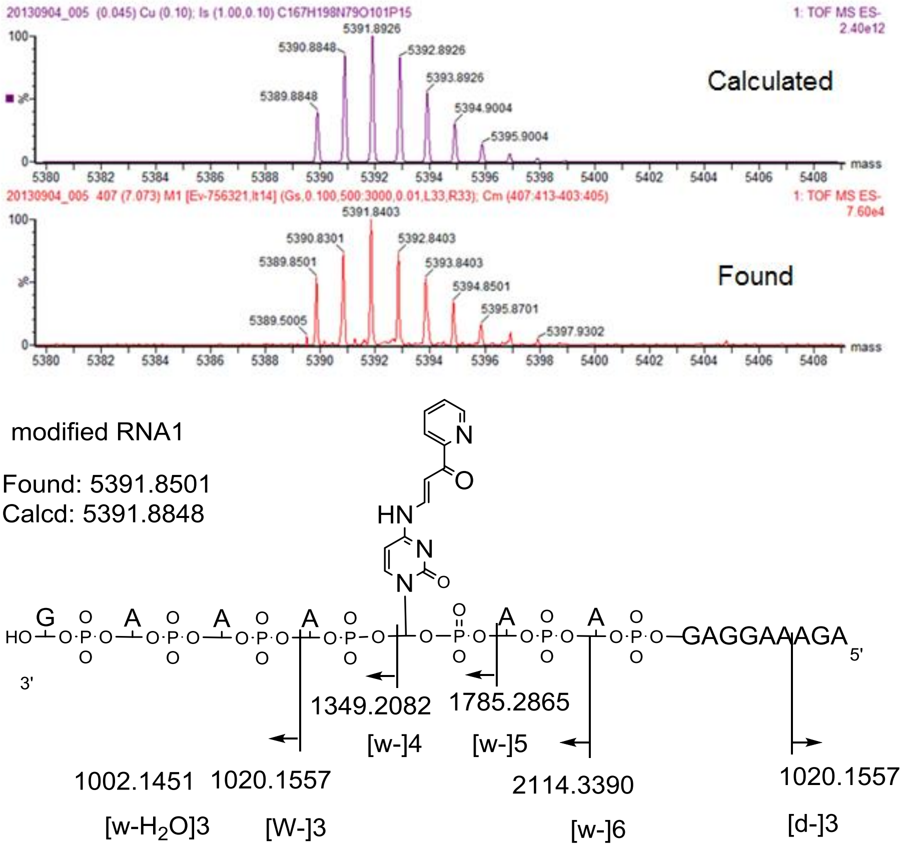

Figure S4. Analysis of MS/MS data of modified RNA**1**.

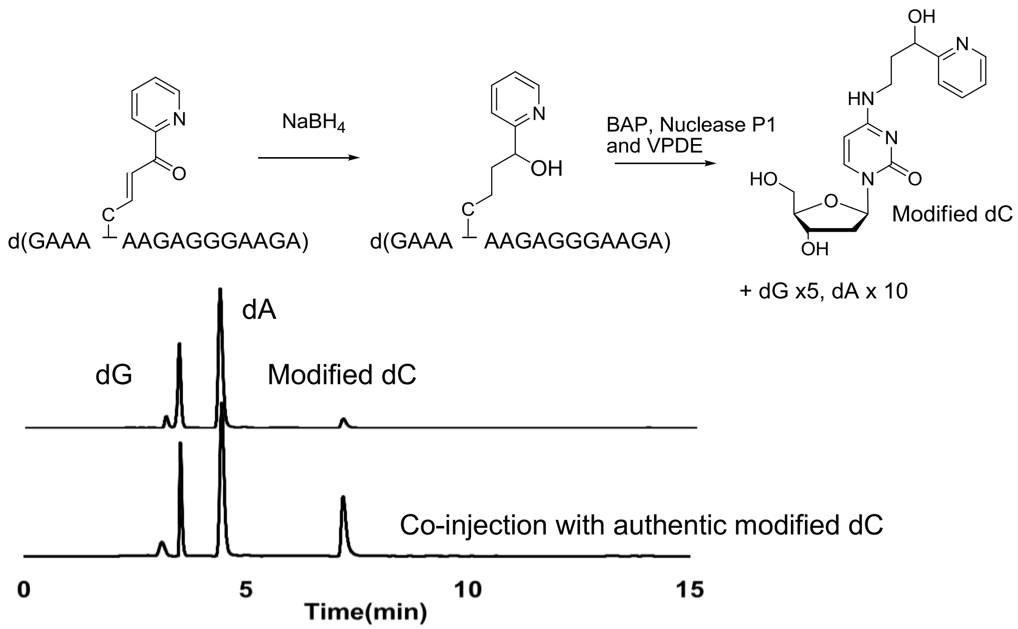

Figure S5. Confirmation of the 4-NH_2 modification of cytosine. The transfer reaction was performed using the DNA substrate with the same sequence with RNA**1**.

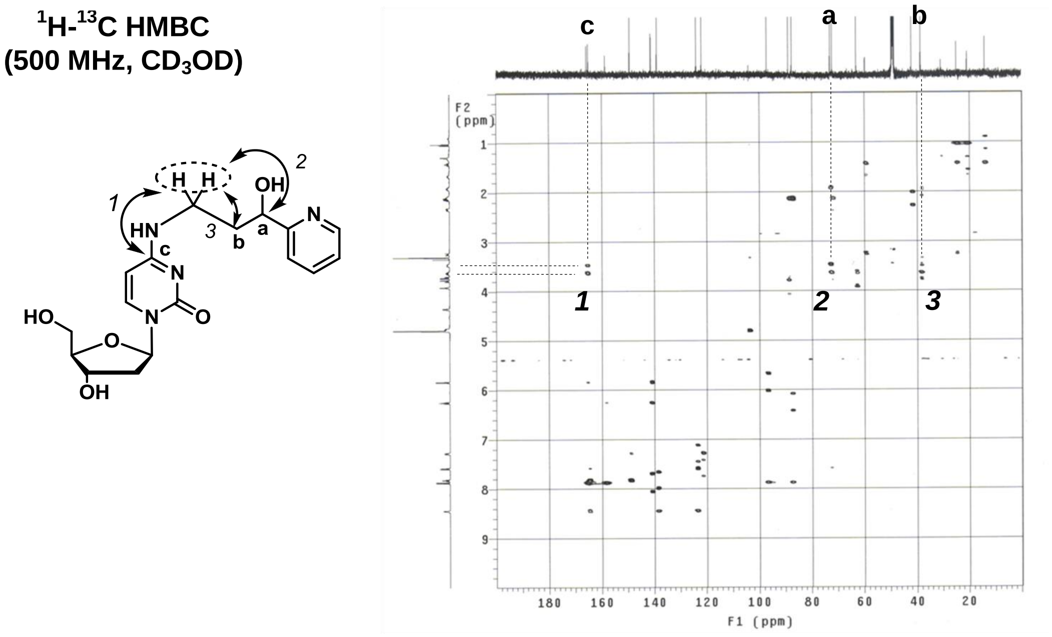

Figure S6. ^1H-^{13}C HMBC spectra of **S3-4** as the authentic sample for the modified C.

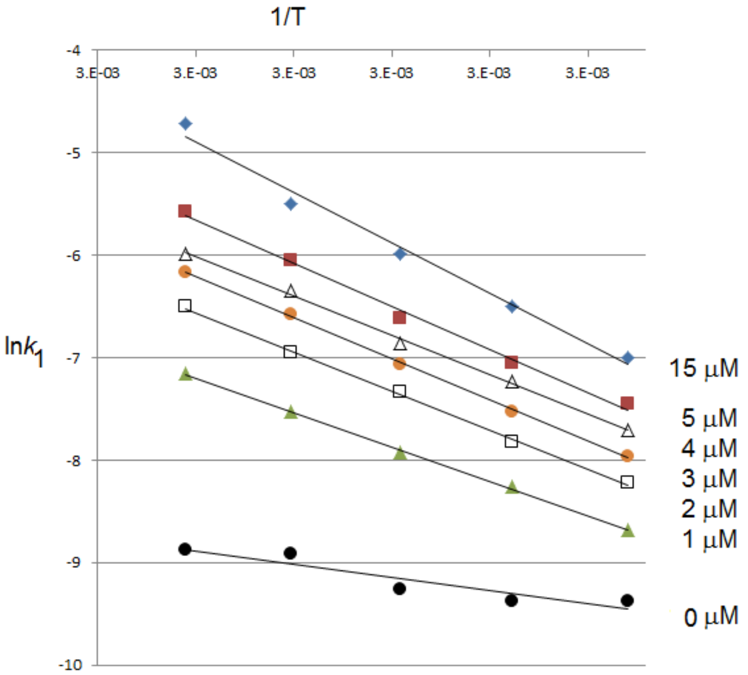

Figure S7. Summary of Arrhenius plots of the transfer reaction.

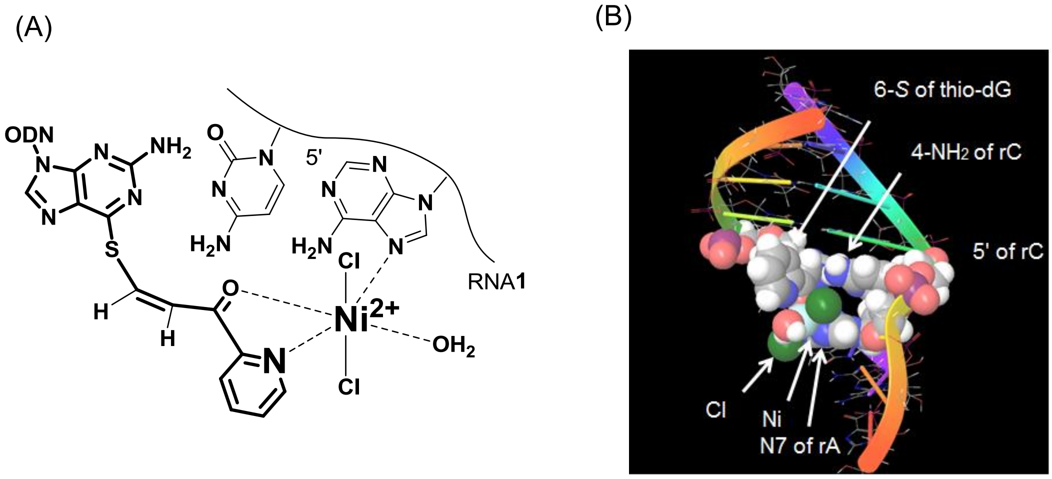

Figure S8. Illustration of a bridging complex with (*Z*)-pyridyl vinyl keto unit, (A) A complex structure formed with $NiCl_2$ and the adenine residue at 5´ side. (B) An optimized structure having a constrained Ni-N7 bond. A distortion of the phosphate backbone was suggested, nonetheless, the vinyl reactive site and 4-amino group of rC were not in close proximity.

Experimental

Preliminary experiments using FT-ODN (1) prepared using the pyridinyl ethynyl keto derivative (5). The modification of 6-thio position of ODN (**1**) was performed using 100 μM of ODN**1** and 500 μM of the alkylating agent (**5**) in 25 mM carbonate buffer at pH 10 and r.t. for 10 min. After dilution of the mixture, the transfer reaction was performed using 6 μM of FT-ODN**1,** 5 μM of RNA**1,** 50 mM HEPES buffer, 100 mM NaCl, 0.6 μM $NiCl_2$ at pH 7.4 and 37 °C. The reaction progress of the modification of ODN**1** and the transfer reaction to RNA**1** (rC) were followed by HPLC (Figure S1). HPLC conditions; column: SHISEIDO C18, 4.6 x 250 mm, solvents, A: 0.1M TEAA, B: CH_3CN, B 10 % to 30 % /20 min, 30 % to 100 % /25 min, linear gradient; flow rate at 1.0 ml/min, UV monitored at 254 nm.

Model study using 6-thio-2'-deoxyguanosine (6-thio-dG) and determination of (*E*)- and (*Z*)-isomer by ^{1}H-NMR

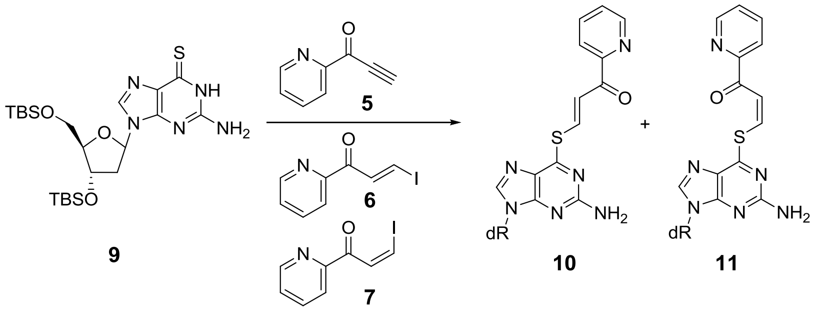

The compound **9** was synthesized according to the literature.[1] IR 3297, 3163, 2954, 2929, 2858, 1650, 1603, 1582, 1555, 1390, 1257, 1195 cm^{-1}. ^{1}H-NMR (400MHz, $CDCl_3$) δ (ppm) 12.6 (1H, bs), 8.28 (1H, s), 6.22 (1H, dd, *J* = 6.7, 6.3 Hz), 5.97 (2H, s), 4.55 (1H, ddd, *J* = 6.1, 3.5, 3.4 Hz), 3.97 (1H, dt, *J* = 4.0, 3.4 Hz), 3.75 (2H, d, *J* = 4.0 Hz), 2.50 (1H, ddd, *J* = 13.1, 6.7, 6.1 Hz), 2.34 (1H, ddd, *J* = 13.1, 6.3, 3.5 Hz), 0.89 (9H, s), 0.87 (9H, s), 0.09 (6H, s), 0.05 (3H, s), 0.05 (3H, s). ^{13}C-NMR (125 MHz, $CDCl_3$) δ (ppm) 175.0, 153.0, 147.7, 140.0, 130.0, 88.1, 83.8, 72.2, 63.1, 40.8, 26.1 (3C), 26.0 (3C), 18.5, 18.1, -4.5, -4.6, -5.2, -5.3. HR-ESI/MS (*m/z*) calcd for $C_{22}H_{42}N_5O_3SSi_2^+$ $[M+H]^+$, 512.2541; found 512.2530. m.p. >300 °C.

A solution of **6** (12 mg, 0.047 mmol) in MeOH (0.4 mL) was added to a solution of **9** (20 mg, 0.039 mmol) and triethylamine (16 µL, 0.118 mmol) in MeOH (0.4 mL) under an argon atmosphere at room temperature. After stirring for 30 min, the mixture was diluted with $CHCl_3$ (10 mL), washed saturated aqueous NH_4Cl (10 mL). The aqueous phase was extracted with $CHCl_3$ (10 mL×2). The combined organic phase was washed with brine (10 mL), dried over Na_2SO_4, and evaporated. The residue was chromatographed on a silica gel column (FUJI SYLISIA FL60D, 5 g, Hex-AcOEt = 1:1, v/v) to give **10** and **11** as yellow viscous oil (22 mg, 0.033 mmol, 86 %). The *E*- to *Z*-ratio (**10/11**) was determined by ^{1}H-NMR (Figure S2).

Mixture of **10** and **11**: IR 3320, 3195, 2952, 2930, 2896, 2858, 1660, 1595, 1546, 1507, 1462 cm^{-1}. ^{1}H-NMR (500MHz, $CDCl_3$) δ (ppm) 9.29 (0.9H, d, *J* = 16.1 Hz), 9.05 (0.1H, d, *J* = 10.0 Hz), 8.71 (1H, ddd, *J* = 4.8, 1.7, 0.9 Hz), 8.17 (1H, ddd, *J* = 7.8, 1.1, 0.9 Hz) 8.10 (0.9H, d, *J* = 16.1 Hz), 8.02 (0.1H, d,

J = 10.0 Hz), 8.02 (0.1H, s), 7.99 (0.9H, s), 7.85 (1H, ddd, *J* = 7.8, 7.6, 1.7 Hz), 7.47 (1H, ddd, *J* = 7.6, 4.8, 1.1 Hz), 6.32 (1H, dd, *J* = 6.8, 6.3 Hz), 5.13 (1.8H, s), 5.09 (0.2H, s), 4.59 (1H, ddd, *J* = 6.1, 3.5, 3.0 Hz), 3.99 (1H, ddd, *J* = 4.1, 3.3, 3.0 Hz), 3.81 (1H, dd, *J* = 11.2, 4.1 Hz), 3.75 (1H, dd, *J* = 11.3, 3.3 Hz), 2.59 (1H, ddd, *J* = 13.1, 6.8, 6.1 Hz), 2.37 (1H, ddd, *J* = 13.1, 6.3, 3.5 Hz), 0.91 (9H, s), 0.90 (9H, s), 0.10 (6H, s), 0.07 (3H, s), 0.07 (3H, s). ^{13}C-NMR (125 MHz, $CDCl_3$) δ (ppm) 189.1 (0.1C), 186.8 (0.9C), 159.0 (0.9C), 158.9 (0.1C), 157.7 (0.1C), 155.6 (0.9C), 153.9 (0.9C), 153.9 (0.1C), 151.8 (0.1C), 151.6 (0.9C), 149.0, 140.8 (0.1C), 140.6 (0.9C), 139.6 (0.1C), 139.6 (0.9C), 137.2, 127.0 (0.1C), 126.9 (0.1C), 126.8 (0.1C), 126.3 (0.9C), 123.2(0.9C), 123.1 (0.1C), 121.0 (0.9C), 117.8 (0.1C), 88.0 (0.9C), 87.9 (0.1C), 83.9 (0.9C), 83.8 (0.1C), 72.2 (0.9C), 72.1 (0.1C), 63.0, 41.0 (0.9C), 41.0 (0.1C), 26.1 (3C), 25.9 (3C), 18.6, 18.1, -4.5, -4.6, -5.2, -5.4. HR-ESI/MS (*m/z*) calcd for r $C_{30}H_{47}N_6O_4SSi_2^+$ $[M+H]^+$, 643.2913; found 643.2896.

Click reaction of modified ORN1 (Py-acetylene) with biotin-N_3 in the presence of $CuSO_4$ as a general procedure for click reaction

ODN**1** was functionalized with **8** to produce FT-ODN-**S1** bearing the acetylene-pyridinyl keto unit, which was subjected to the functionality transfer reaction with RNA**1**(rC) as described above. A solution of the py-acetylene-modified ORN**1**(rC) (5 µM, 20 µL, 100 pmol), biotin-N_3 (25 mM, 0.4 µL, 10 nmol), sodium ascorbate (25 mM, 0.16 µL, 4 nmol), TBTA (25 mM, 0.16 µL, 4 nmol) and $CuSO_4$ (10 mM, 0.2 µL, 2 nmol) were mixed and diluted with DMSO to the volume of 25 µL (final concentrations: ORN**17,** 4 µM; biotin-N_3, 400 µM; sodium ascorbate, 160 µM; TBTA, 160 µM; $CuSO_4$, 80 µM). The mixture was incubated at 37 °C for 30 min, and analyzed by HPLC (Column: SHISEIDO C18, TYPE MG, 4.6×250 mm; Solvent: A: 0.1 M TEAA Buffer, B: CH_3CN, B: 10 % to 30 % /20 min, 30 % to 100 % /25 min, linear gradient; flow rate, 1.0 mL/min; monitored by UV detector at 254 nm).

Confirmation of the modified position of RNA1

The modified **RNA1** was isolated and subjected to MS/MS analysis using the following conditions. An Acquity UPLC H-Class TUV system (Waters, Milford, MA, USA) fitted with an Acquity BEH C18 column (2.1 x 150 mm; 1.7 µm); Mobile phase A = 15 mM TEA, 400 mM HFIP in water; Mobile phase B = 1:1 methanol:A; Linear gradient, B % = 35>45>35>35 (0.0>14.0>14.1>20.0 min); Flow rate, 0.2 mL/min; Column temperature, 60 °C; UV, monitored at 254 nm. MS/MS was measured by Xevo G2-S QToF system (Waters, Manchester, UK) using ESI negative ion mode; capillary and cone voltage of 3.0 kV and 30 V respectively; heated gas flow (300 °C) of 1200 L/hr; Ionization temperature, 120 °C. The $[M-4H]^{-4}$ at *m/z* 1346 eluting in the peak at 7.04 min and corresponding to the modified RNA**1** was subjected MS/MS measurements. Data from the region of interest are summarized in Figure S4, clearly indicating that rC is modified as expected.

Determination of the structure of the product of the functionality transfer reaction

To determine the structure of the modified cytidine, the transfer reaction was performed using the corresponding DNA**1**, 5' AGAAAGGAGAA-**C**-AAAG., in which rC represents the target dC. The reaction was performed using 15 µM of (*E*)-FT-ODN**1** and 10 µM of DNA**1** in the buffer 50 mM HEPES and 100 mM NaCl, 1 mM $NiCl_2$ at pH 7 and 37°C. The modified DNA substrate was purified,

freeze-dried, and subjected to reduction in a carbonate buffer (25 mM, pH 10) containing 100 mM $NaBH_4$ for 30 min at room temperature. The reaction mixture was neutralized with acetic acid and purified by HPLC. The reduced DNA substrate was diluted with ten-times diluted BAP buffer, followed by the addition of bacterial alkaline phosphatase (BAP, 0.05 u/μL), nuclease P1 (0.08 u/μL) and venom phosphodiesterase (VPDE, 0.01 u/μL). The mixture was incubated for 60 min at 37 °C, and analyzed by HPLC using the following conditions (Figure S5). HPLC conditions: column, SHISEIDO CAPCELL PAK C18, TYPE MG; flow rate: 1 mL/min; solvent A = 50 mM $HCOONH_4$, solvent B = CH_3CN, 10 % to 55 % /20 min, 55 % to 100 % /25 min, linear gradient, monitored at 254 nm. The peak corresponding to the modified dC was confirmed by ESI-MS and comparison by HPLC co-injection with the authentic sample (Figure S5).

Synthesis of the authentic sample of the modified-dC

3-Oxo-3-(pyridin-2-yl)propanenitrile[2,3]

A solution of ethyl 2-picolinate (2.68 mL, 19.8 mmol) and CH_3CN (1 mL, 19.8 mmol) in toluene (10 mL) was slowly added to a solution of NaH 60 % oil suspension (794 mg, 19.8 mmol) in toluene (50 mL) at 65 °C under an argon atmosphere. After stirring at 65 °C for 14.5 h, the reaction mixture was cooled down, and diluted with ice water (20 mL). The aqueous phase was washed with diethyl ether and neutralized with 10 % aqueous HCl to form brown precipitates. The precipitates were collected, washed with water and dried over under reduced pressure to give the title compound as a brown powder (1.66 g, 11.3 mmol, 64 %). IR 1714, 1585, 1439, 1384, 1330 1219, 1013 cm^{-1}. ^{1}H-NMR (400MHz, $CDCl_3$) δ (ppm) 8.67 (1H, dd, *J* = 4.9, 1.2 Hz), 8.09 (1H, dd, *J* = 7.9, 1.2 Hz), 7.88 (1H, ddd, *J* = 7.9, 7.6, 1.2 Hz), 7.55 (1H, ddd, *J* = 7.6, 4.9, 1.2 Hz), 4.36 (2H, s). HR-ESI/MS (*m/z*) calcd for $C_8H_7N_2O^+$ $[M+H]^+$, 147.06; found 147.07. mp. 95 °C

3-Amino-1-(pyridin-2-yl)propan-1-ol (S3-2)

$LiAlH_4$ (657 mg, 17.11 mmol) was added into a solution of the above product (500 mg, 3.42 mmol) in THF (30 mL) at 0 °C under an argon atmosphere. The reaction mixture was heated to 80 °C under reflux. After 4 h, the reaction mixture was cooled to 0 °C, followed by the addition of water (3 mL) and 10 % aqueous NaOH (1.5 mL). The resulting precipitates were filtrated through a Celite pad and the filtrate was evaporated to dryness to give **S3-2** as a brown foam (494 mg). The product was used for next step without further purification.

Synthesis of the authentic adduct (S3-4)

S3-2 (126 mg) was added into a solution of **S3-3** (202 mg, 0.277 mmol) in MeOH (3 mL) at room temperature. After stirring at room temperature for 1.5 h, the solvent was removed under reduced pressure to give a brown crude product, which was purified by flash column chromatography (chromatography was done twice, 1st; FUJI SYLISIA FL60D, 15 g, $CHCl_3$-MeOH = 1:0 - 10:1, v/v. 2nd; FUJI SYLISIA FL60D, 15 g, $CHCl_3$-MeOH = 1:0 - 99:1, v/v) to give the TBS protected derivative of **S3-4** as a pale yellow foam (31 mg, 0.052 mmol, 19 %). A solution of the above product (24 mg, 0.0399 mmol) in THF (1 mL) and TBAF in THF (1 M, 0.1 mL, 0.0998) was stirred at room temperature under an argon atmosphere. After stirring at room temperature for 35 min, the solvent was removed under

reduced pressure. The residue was purified by flash column chromatography (FUJI SYLISIA FL60D, 4 g, $CHCl_3$-MeOH =5:1, v/v) to give a pale yellow product, which was further purified by HPLC to give **S3-4** as a white foam (14 mg, 0.0388 mmol, 94 %). IR 3288, 2934, 1645, 1571, 1509, 1474, 1436, 1323, 1287, 1197, 1095, 1057 cm^{-1}. ^{1}H-NMR (400MHz, CD_3OD) δ (ppm) 8.44 (1H, d, *J* = 5.0 Hz), 7.87 (1H, d, *J* = 7.3 Hz), 7.82 (1H, ddd, *J* = 7.9, 7.2, 1.5 Hz), 7.58 (1H, d, *J* = 7.9 Hz), 7.27 (1H, dd, *J* = 7.2, 5.0 Hz), 6.24 (1H, t, *J* = 6.3 Hz), 5.83 (1H, d, *J* = 7.3 Hz), 4.75 (1H, dd, *J* = 8.9, 3.4 Hz), 4.35 (1H, dt, *J* = 6.7, 3.7, 3.1 Hz), 3.91 (1H, ddd, *J* = 3.8, 3.7, 3.4 Hz), 3.77 (1H, dd, *J* = 12.2, 3.4 Hz), 3.70 (1H, dd, *J* = 12.2, 3.8 Hz), 3.66-3.58 (1H, m), 3.49-3.42 (1H, m), 2.32 (1H, ddd, *J* = 13.4, 6.7, 6.3 Hz), 2.15-2.05 (2H, m), 1.94-1.85 (1H, m). ^{13}C-NMR (125 MHz, CD_3OD) δ (ppm) 165.6, 164.9, 158.5, 149.2, 141.1, 138.8, 123.7, 121.7, 97.0, 88.8, 87.5, 72.8, 72.1, 62.8, 41.9, 38.4(2C). HR-ESI/MS (*m/z*) calcd for $C_{17}H_{23}N_4O_5^+$ $[M+H]^+$, 363.1628; found 363.1663.

Kinetic analysis of the functionality transfer reaction using RNA1(rC) and (*E*)-FT-ODN1

The reaction within the DNA/RNA duplex was analyzed as the first-order reaction using the initial duplex concentration of 4.5 μM as the reactive duplex formed with (*E*)-FT-ODN**1**, and the rest (0.5 μM) as the nonreactive one formed with (*Z*)-FT-ODN**1.** The HPLC peak of rC-modified RNA**1** was quantified and the half-life ($t_{1/2}$ *s*) of the reaction was obtained, then the first-order rate constant (k_1) was calculated by the equation (1). The k_1 values were obtained at the different temperature (15, 20, 25, 30 and 35°C) and in the presence of different concentrations of $NiCl_2$ (0, 1, 2, 3, 4, 5, 15 μM). The obtained rate constants (k_1) were subjected to Arrhenius plot, and the E_a value was obtained by the equation (2). $\Delta G^‡$, $\Delta H^‡$ and $\Delta S^‡$ were obtained by the Eyring equation (3)-(7). Figure S7 summarizes the Arrhenius plots. Table S2 summarizes the kinetic parameters, which are expressed in the bar graph in Figure 5.

k_1=ln*2/* $t_{1/2}$ (1)

$k_{1:}$ the first-order rate constant, $t_{1/2}$: half-life

lnk_1=-E_a/RT+lnA (2)

E_a: activation energy, *R*: gas constant, R = 8.314 J K^{-1} mol^{-1}

$K^‡$=(*h* k_1)/(k_B T) (3)

h: the Planck constant, *h* = 6.626 x 10^{-34} J·s,

k_B: Boltzmann's constant, k_B = 1.381)×10^{-23} J·K^{-1}

$H^‡$= E_a-RT (4)

$\Delta G^‡$=-RTln($K^‡$) (5)

$\Delta G^‡$=$\Delta H^‡$-T$\Delta S^‡$ (6)

$\Delta S^‡$=($\Delta H^‡$-$\Delta G^{‡)}$/T (7)

Table S1. MALDI-TOF/MS data

Sequence	ODN or RNA	Pyk modifying agent	M^5 or N^5	M^3 or N^3	Calcd ($[M-H]^-$)	Found
5’ CTTT-SG-TTCTCCTTTCT	ODN**1**				4767.76	4767.85
5’ CTTT-(Pyk)SG-TTCTCCTTTCT	FT-ODN**1**	**5**			4898.80	4899.17
5’ CTTT-(Pyk)SG-TTCTCCTTTCT	FT-ODN**1**	**6**			4898.80	4898.76
5’ CTTT-(Pyk)SG-TTCTCCTTTCT	FT-ODN**1**	**7**			4898.80	4898.82
5’ CTTT-(Pyk)SG-TTCTCCTTTCT	FT-ODN**1**	**8**			4922.80	4926.68
5’ CTTM^5-SG-M^{3}TCTCCTTTCT	ODN**2**		dA	dA	4785.78	4785.34
			dA	dG	4801.78	4803.13
			dA	dC	4761.77	4761.34
			dA	T	4776.77	4776.11
			dG	dA	4801.78	4801.72
			dG	dG	4817.77	4817.19
			dG	dC	4777.77	4778.90
			dG	T	4792.77	4793.26
			dC	dA	4761.77	4757.05
			dC	dG	4777.77	4776.46
			dC	dC	4737.76	4737.28
			dC	T	4752.76	4751.00
			T	dA	4776.77	4776.32
			T	dG	4792.77	4791.08
			T	dC	4752.76	4754.66
			T	T	4767.76	4767.85
		**6**	dA	dG	4932.81	4932.47
		**6**	dA	dC	4892.81	4892.02
		**6**	dA	T	4907.81	4907.78
		**6**	dG	dA	4932.81	4932.65
		**6**	dG	dG	4948.81	4948.97
		**6**	dG	dC	4908.80	4910.64
		**6**	dG	T	4923.80	4924.74
		**6**	dC	dA	4892.81	4892.08
		**6**	dC	dG	4908.80	4910.53
		**6**	dC	dC	4868.80	4868.80
		**6**	dC	T	4883.80	4883.10
		**6**	T	dA	4907.81	4907.58
		**6**	T	dG	4923.80	4923.05
		**6**	T	dC	4883.80	4882.76

Table S1. MALDI-TOF/MS data (continued).

5' CTTM^5-SG-M^{3}TCTCCTTTCT	ODN**2**	**6**	T	T	4898.80	4898.76
5' agaaaggagaa -X-aaag	RNA**1**(rC)				5257.84	5257.72
	RNA**1**(rA)				5281.85	5281.70
	RNA**1**(rG)				5297.85	5297.71
	RNA**1**(U)				5258.83	5258.15
Modified	RNA**1**(rC)	**6**			5388.88	5388.35
Modified	RNA**1**(rA)	**6**			5412.89	5410.74
Modified	RNA**1**(rC)	**8**			5412.88	5415.54
Clicked	RNA**1**(rC)	**8+S7-1**			5884.10	5884.75
Clicked	RNA**1**(rC)	**8+S7-2**			5871.00	5870.37
5' agaaaggagaN^5-c-N^{3}aag All purchased	RNA**2**(rC)		a	a	5257.84	5257.72
			a	g	5273.84	5276.16
			a	c	5233.83	5235.31
			a	u	5234.81	5239.25
			g	a	5273.84	5278.04
			g	g	5289.83	5293.79
			g	c	5249.82	5254.57
			g	u	5250.81	5252.40
			c	a	5233.83	5237.25
			c	g	5249.82	5254.43
			c	c	5209.82	5212.32
			c	u	5210.80	5212.43
			u	a	5234.81	5236.65
			u	g	5250.81	5253.17
			u	c	5210.80	5211.89
			u	u	5211.79	5215.26
			7-deaza-g	g	5288.84	5292.53
			g	7-deaza-g	5288.84	5291.60
			7-deaza-g	7-deaza-g	5287.84	5290.80
	Modified RNA**2**(rC)	**6**	a	a	5388.88	5388.35
		**6**	a	g	5404.87	5402.90
		**6**	a	c	5364.87	5362.34
		**6**	a	u	5365.85	5365.83
		**6**	g	a	5404.87	5404.65
		**6**	g	g	5420.87	5421.29
		**6**	g	c	5380.86	5382.86

Table S1. MALDI-TOF/MS data (continued).

	Modified RNA**2**(rC)	**6**	g	u	5381.85	5381.99
		**6**	c	a	5364.87	5370.60
		**6**	c	g	5380.86	5282.25
		**6**	c	c	5340.86	-
		**6**	c	u	5341.84	-
		**6**	u	a	5365.85	5365.81
		**6**	u	g	5381.85	5382.14
		**6**	u	c	5341.84	-
		**6**	u	u	5342.82	-
		**6**	7-deaza-g	g	5419.87	5421.37
		**6**	g	7-deaza-g	5419.87	5419.94
		**6**	7-deaza-g	7-deaza-g	5418.88	5418.08

Table S2. Kinetic parameters of the functionality transfer reaction.[a]

$NiCl_2$ (μM)	k_1 (25 °C)	E_a(J)	$\Delta G^‡$	±[b]	$\Delta H^‡$	±[b]	$-T\Delta S^‡$ (37 °C)	±[b]
15	2.50E-03	8.45E+04	8.76E+04	3.87E+02	8.21E+04	5.37E+01	5.79E+03	3.34E+02
5	1.34E-03	7.38E+04	8.92E+04	5.87E+02	7.13E+04	6.57E+01	1.86E+04	2.27E+02
4	1.05E-03	6.65E+04	8.99E+04	7.64E+02	6.40E+04	6.57E+01	2.70E+04	1.65E+02
3	8.50E-04	7.08E+04	9.05E+04	6.91E+02	6.83E+04	6.57E+01	2.31E+04	1.84E+02
2	6.50E-04	6.58E+04	9.10E+04	8.01E+02	6.34E+04	6.57E+01	2.90E+04	1.47E+02
1	3.60E-04	5.62E+04	9.26E+04	1.04E+03	5.37E+04	6.57E+01	4.05E+04	9.61E+01
0	9.50E-05	2.72E+04	9.57E+04	2.04E+03	2.47E+04	6.57E+01	7.39E+04	3.26E+02

[a] See pS10 for experimental detail. Figure S7 summarizes Arrhenius plots to obtain E_a values. [b] The standard deviation from the mean of the data obtained at different temperature.

1 K. Onizuka; Y. Taniguchi; S. Sasaki, *Nucleosides, Nucleotides, and Nucleic Acids*, **2009,** *28,* 752-760.

2 Patent: WO2011/126903 A2, **2011** .

3 Christopher, M. Pask.; Kenneth, D. Camm.; Colin, A. Kilner.; Malcolm, A. Halcrow. *Tetrahedron Letters*, **2006**, *47*, 2531–2534

^{13}C-NMR DEPT

5

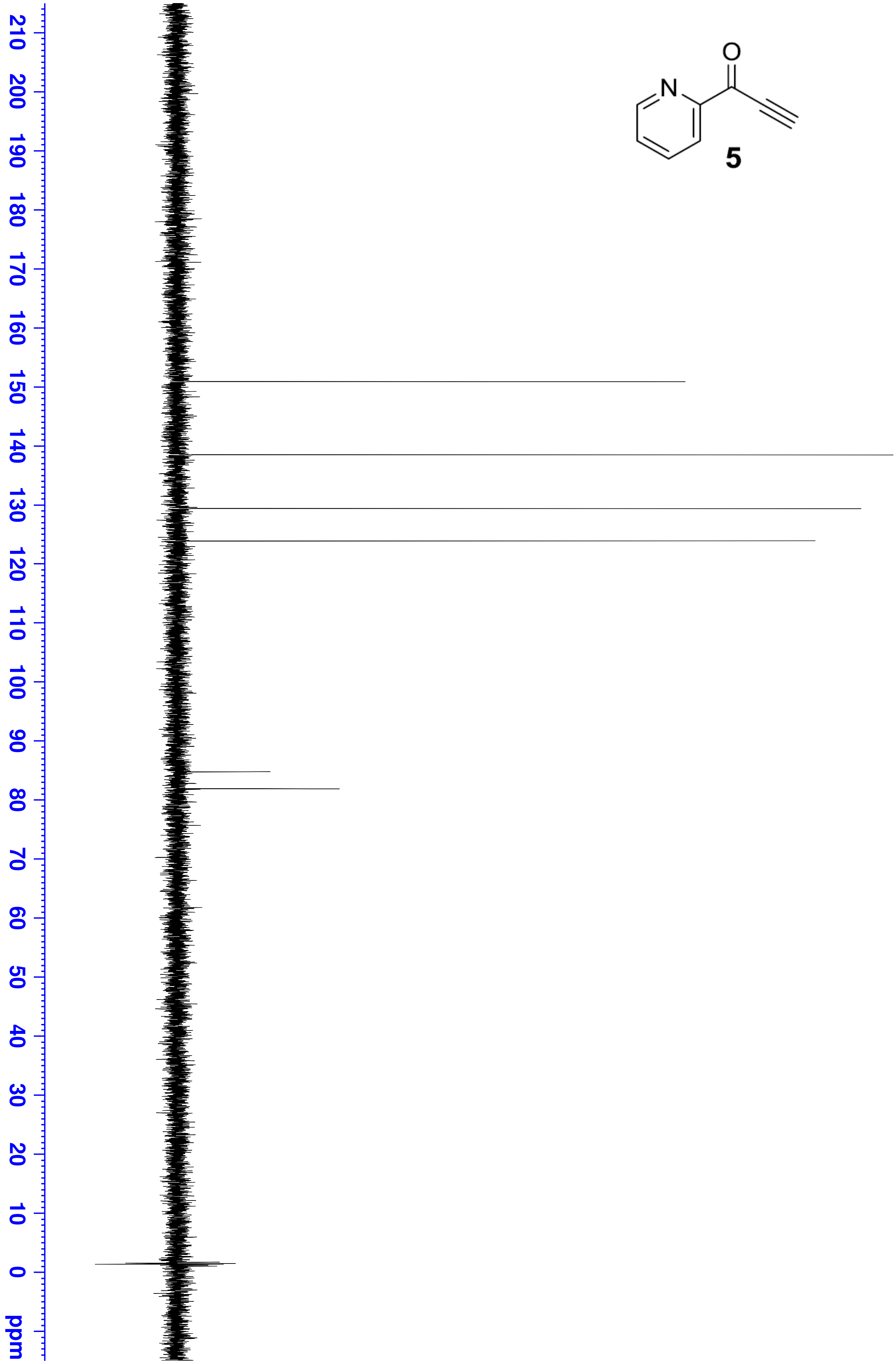

^{13}C-NMR

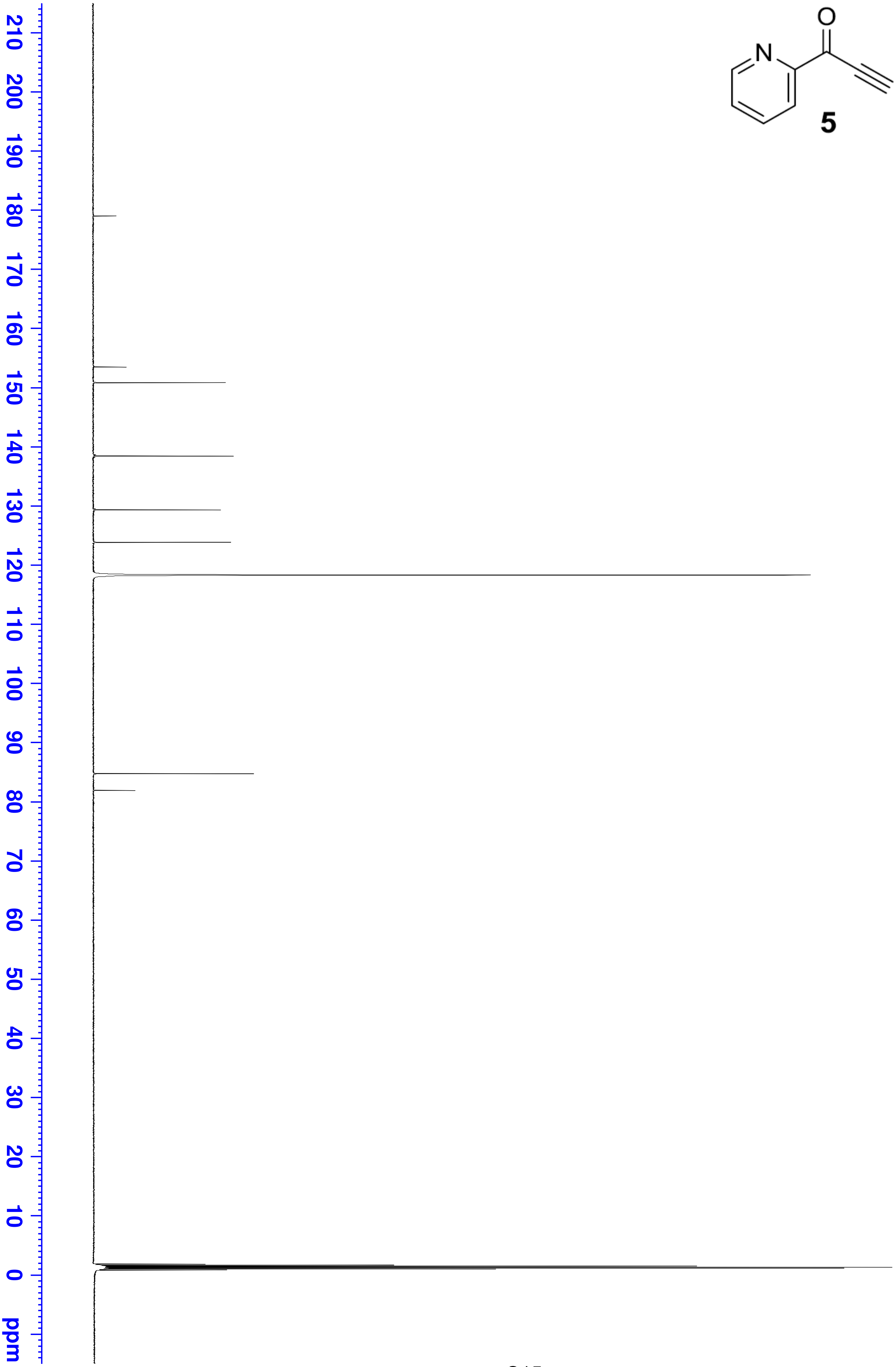
5
210
200
190
180
170
160
150
140
130
120
110
100
90
80
70
60
50
40
30
20
10
0
ppm

^{1}H-NMR

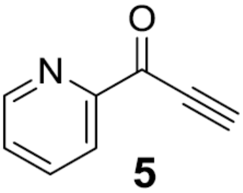

5

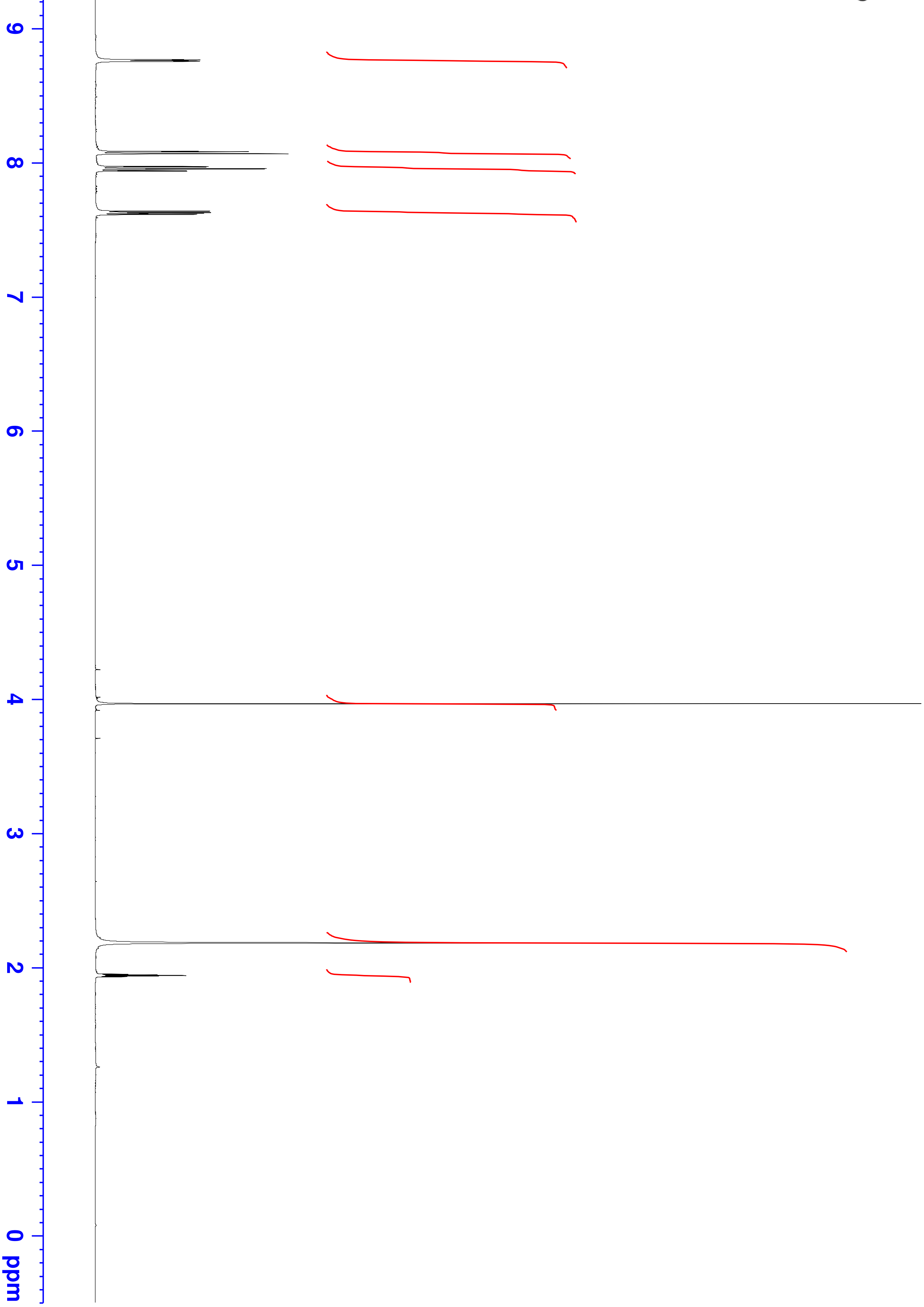

^{13}C-NMR DEPT

6

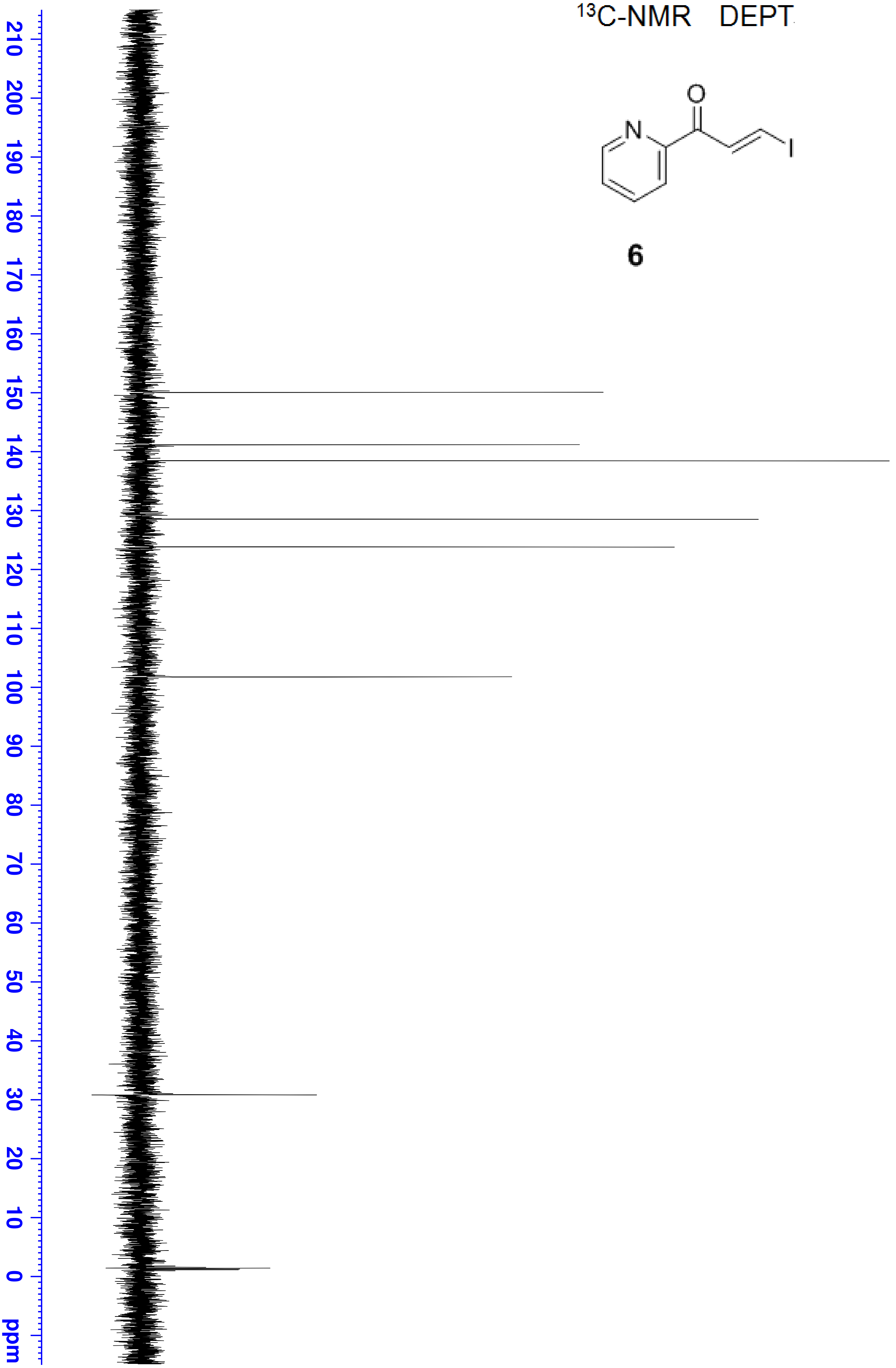

^{13}C-NMR

6

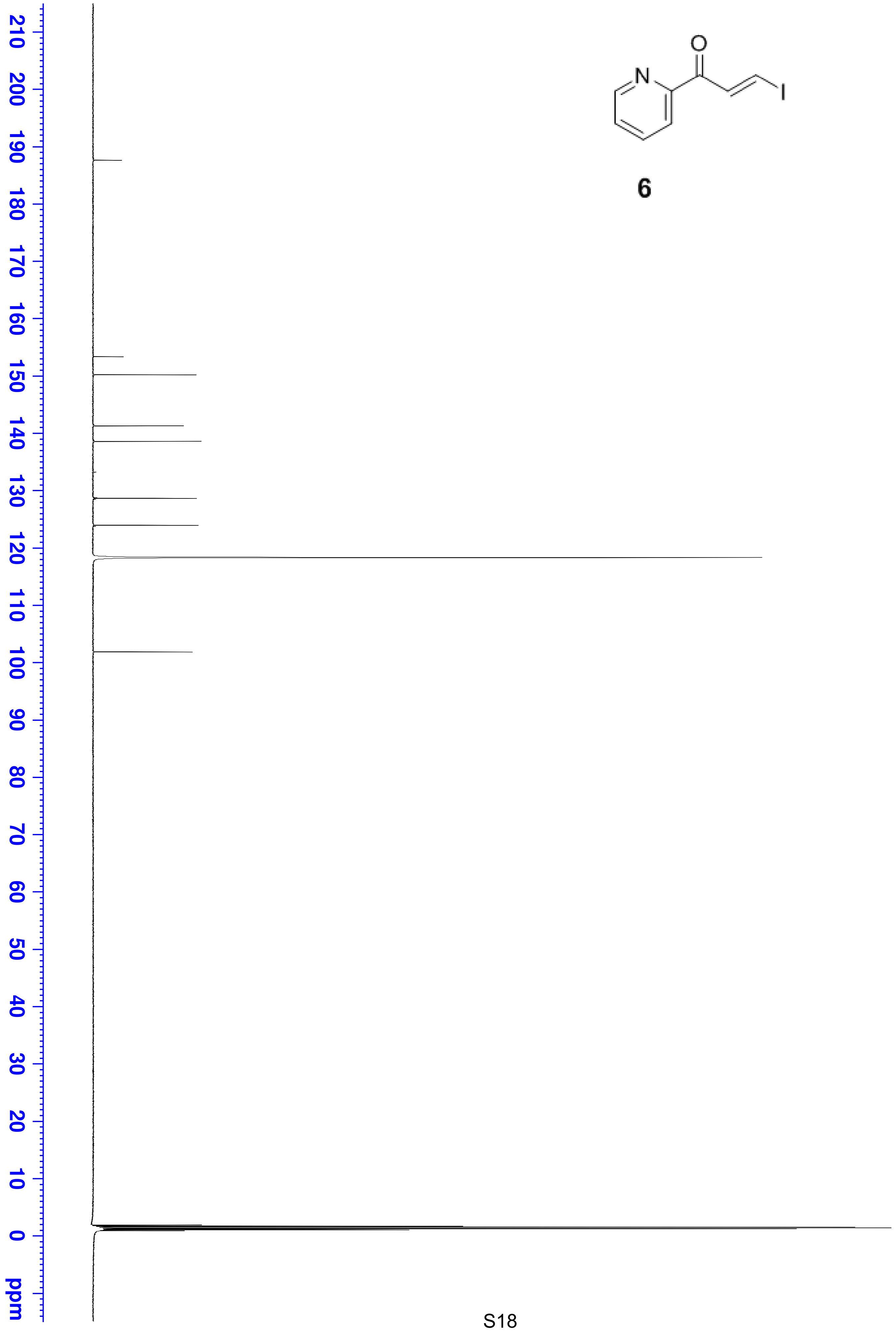

^{1}H-NMR

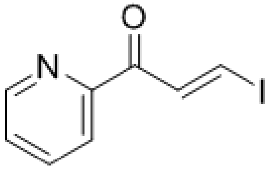

6

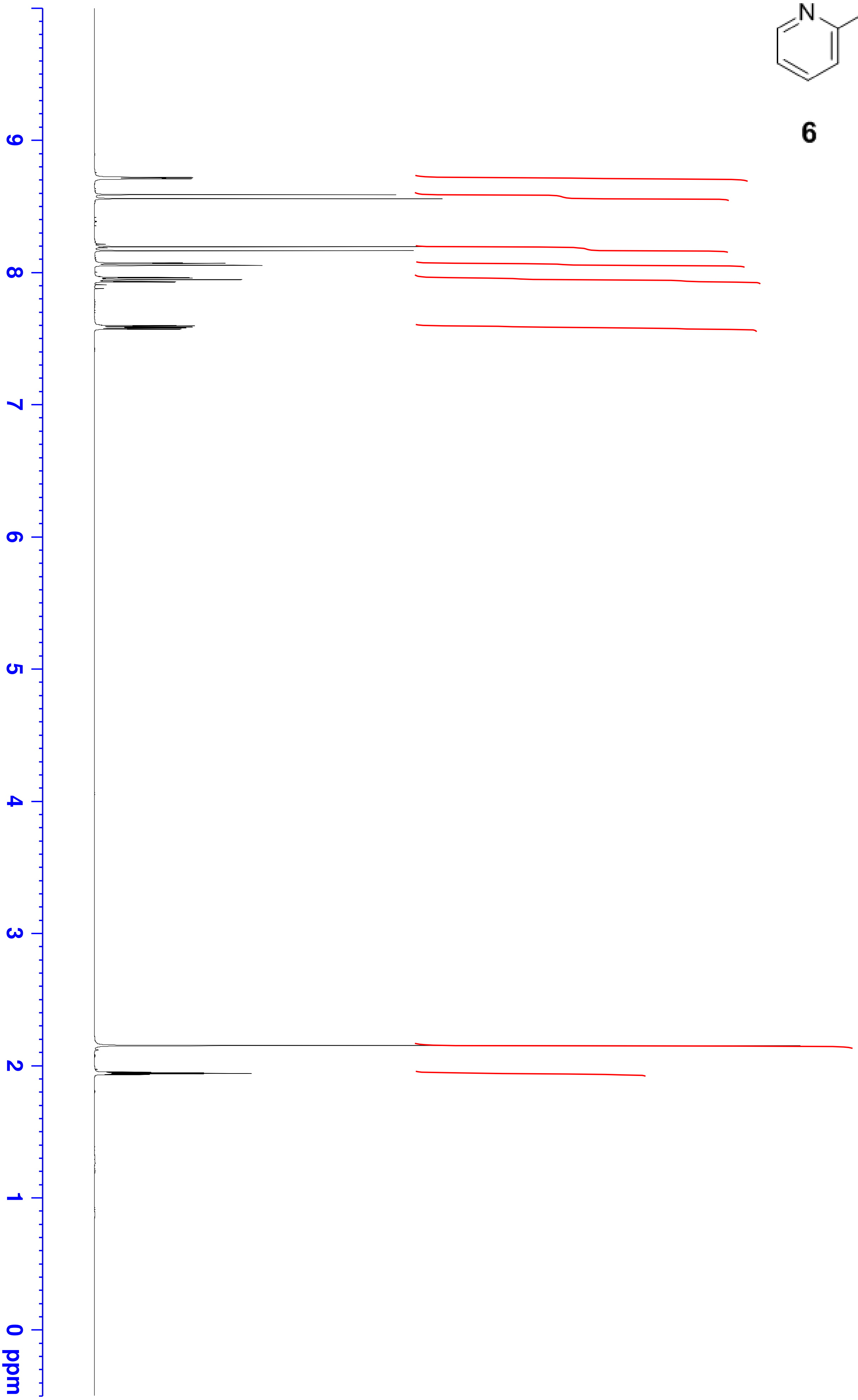

^{13}C-NMR DEPT

7

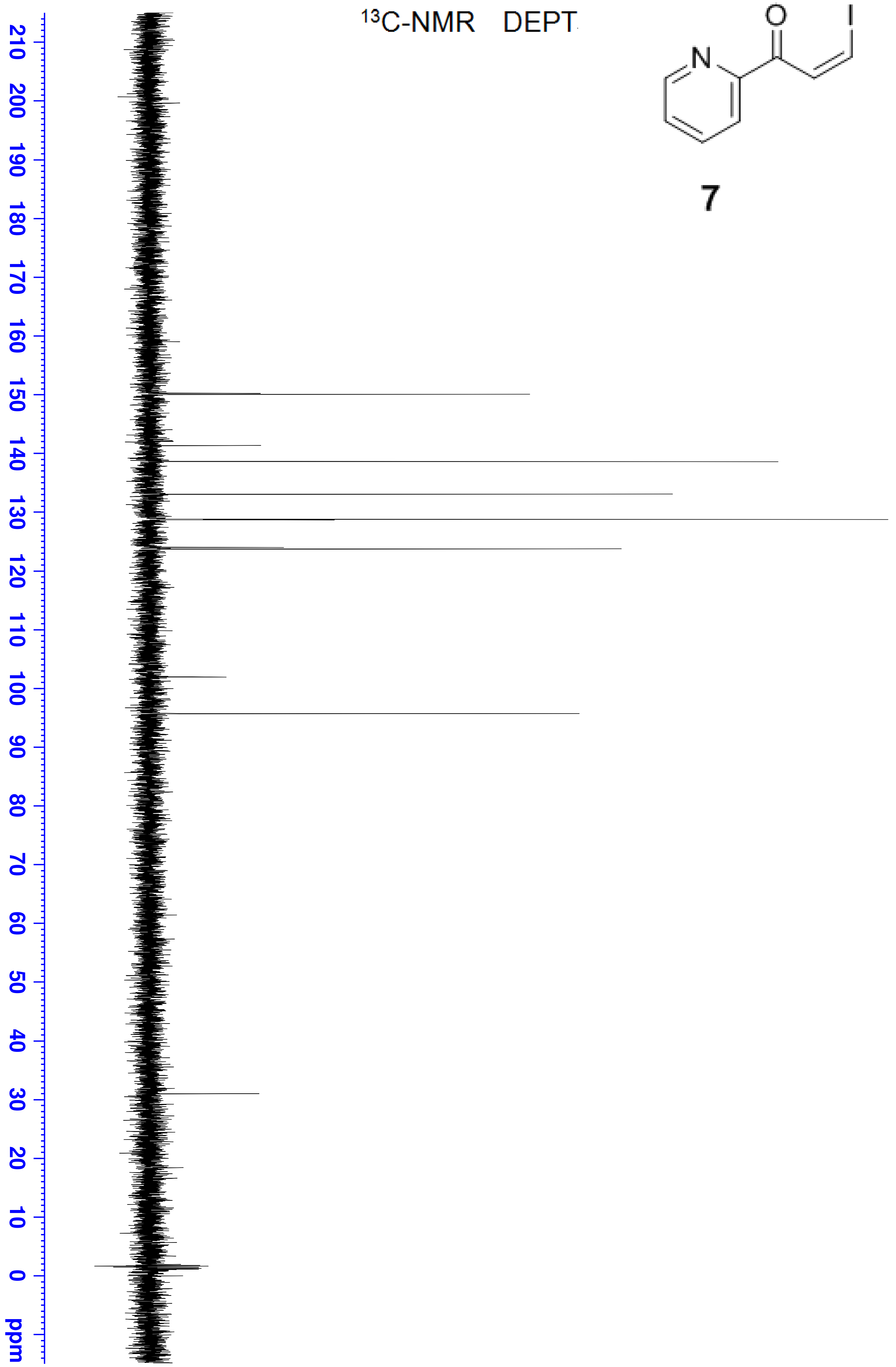

^{13}C-NMR

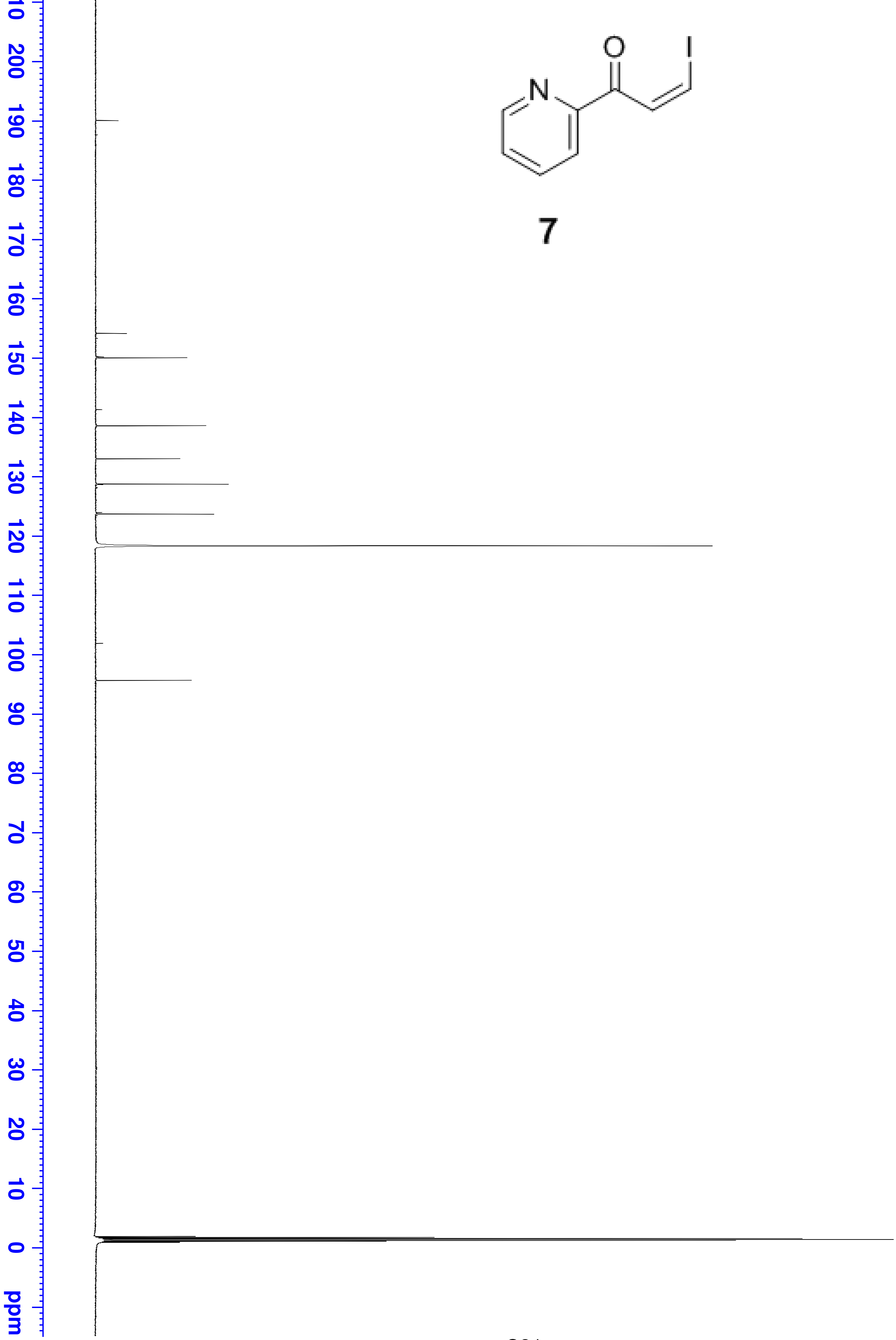

O
N
I
7
210
200
190
180
170
160
150
140
130
120
110
100
90
80
70
60
50
40
30
20
10
0
ppm

^{1}H-NMR

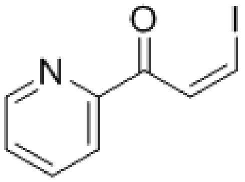

7

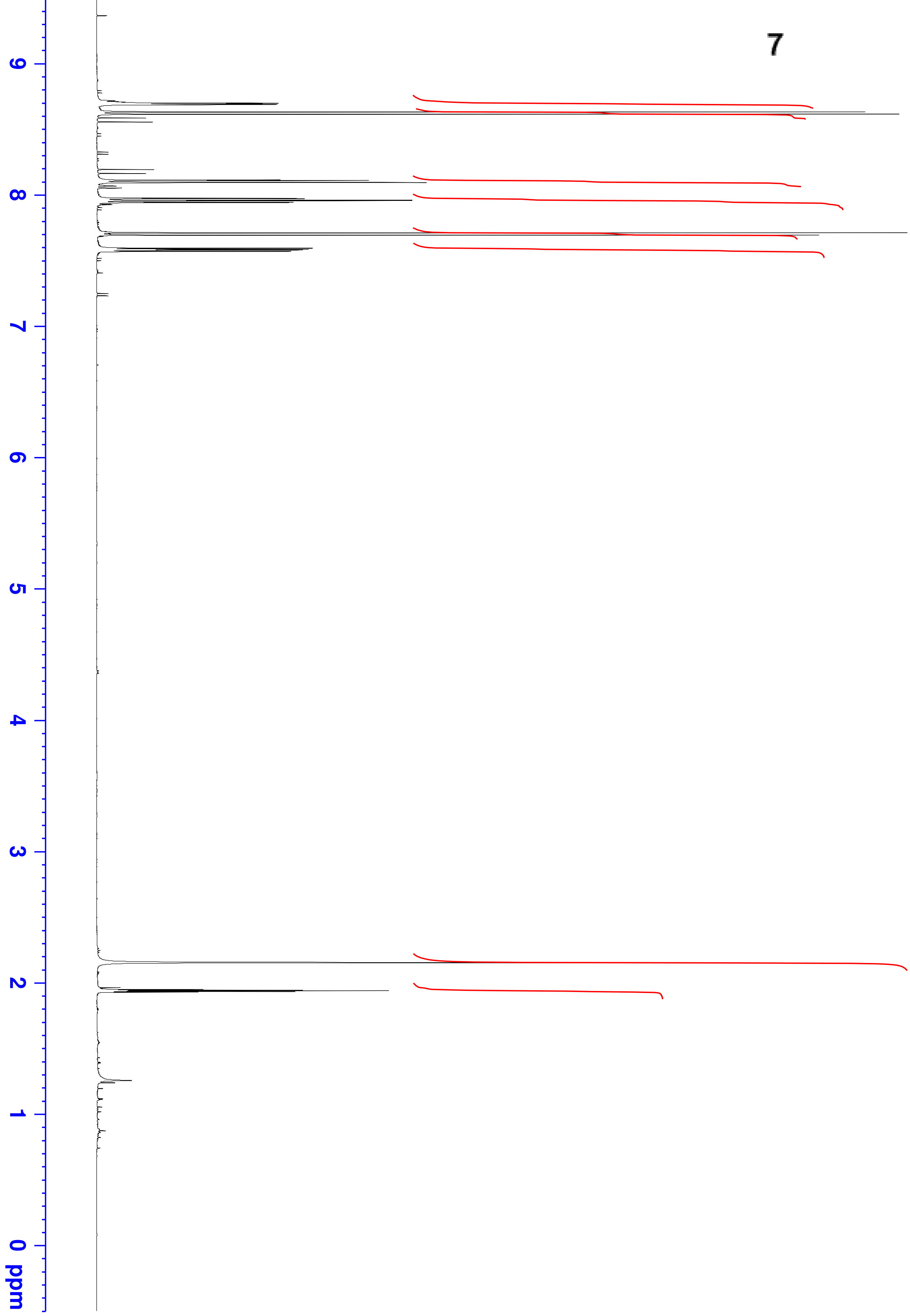

^{13}C-NMR DEPT

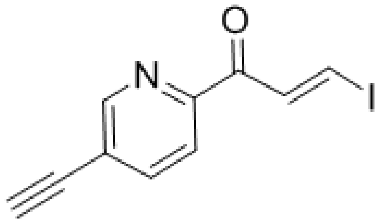

8

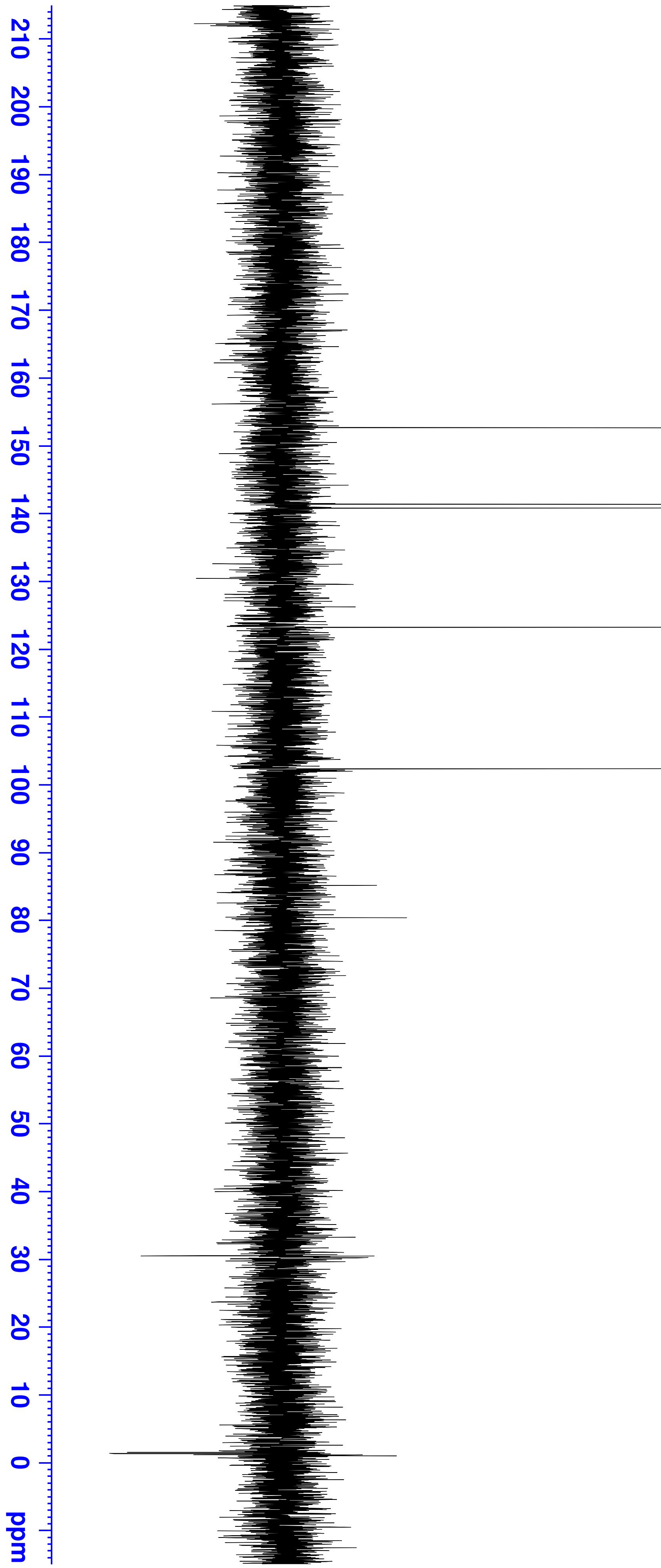

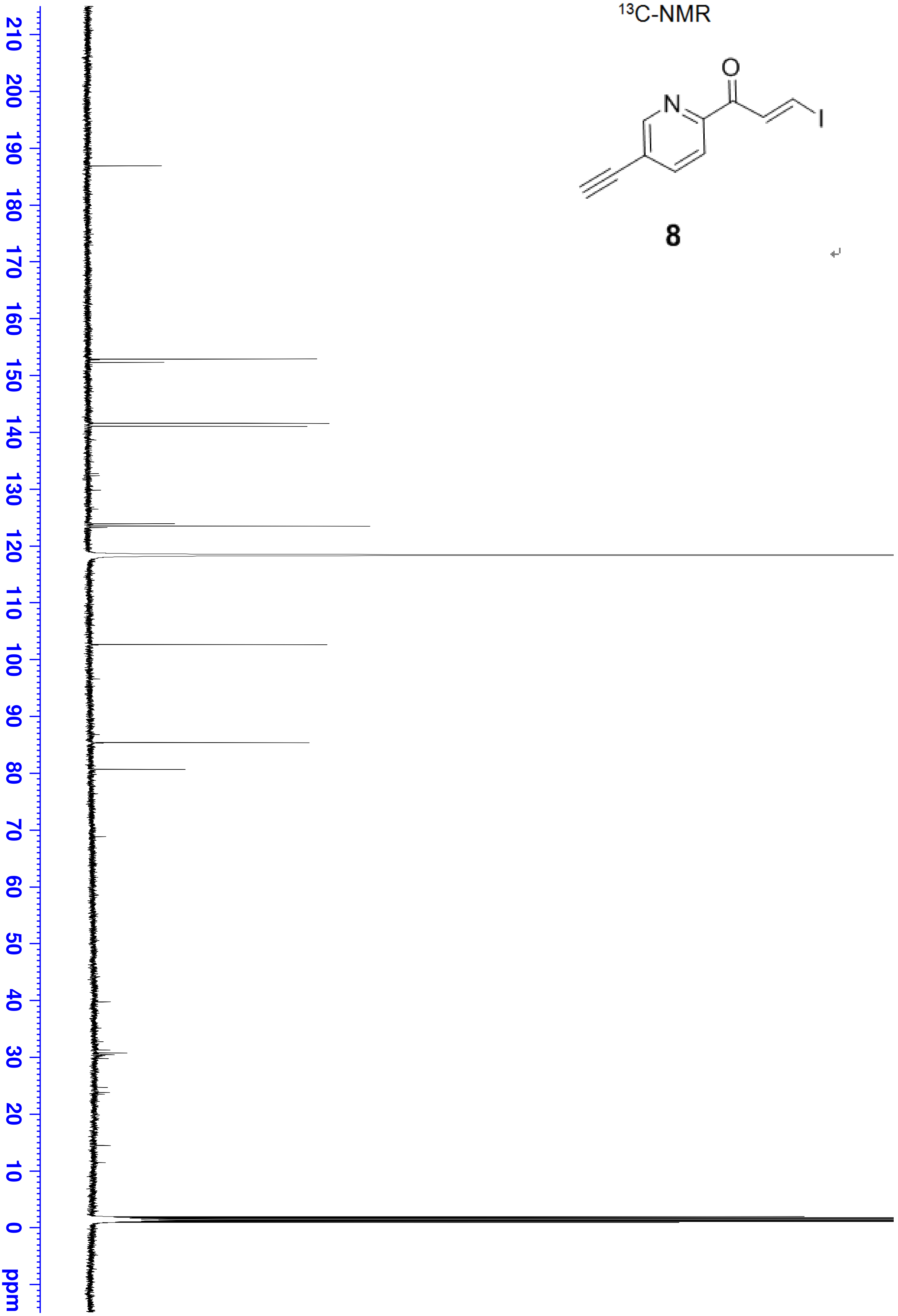

^{13}C-NMR
8
210
200
190
180
170
160
150
140
130
120
110
100
90
80
70
60
50
40
30
20
10
0
ppm

^{1}H-NMR

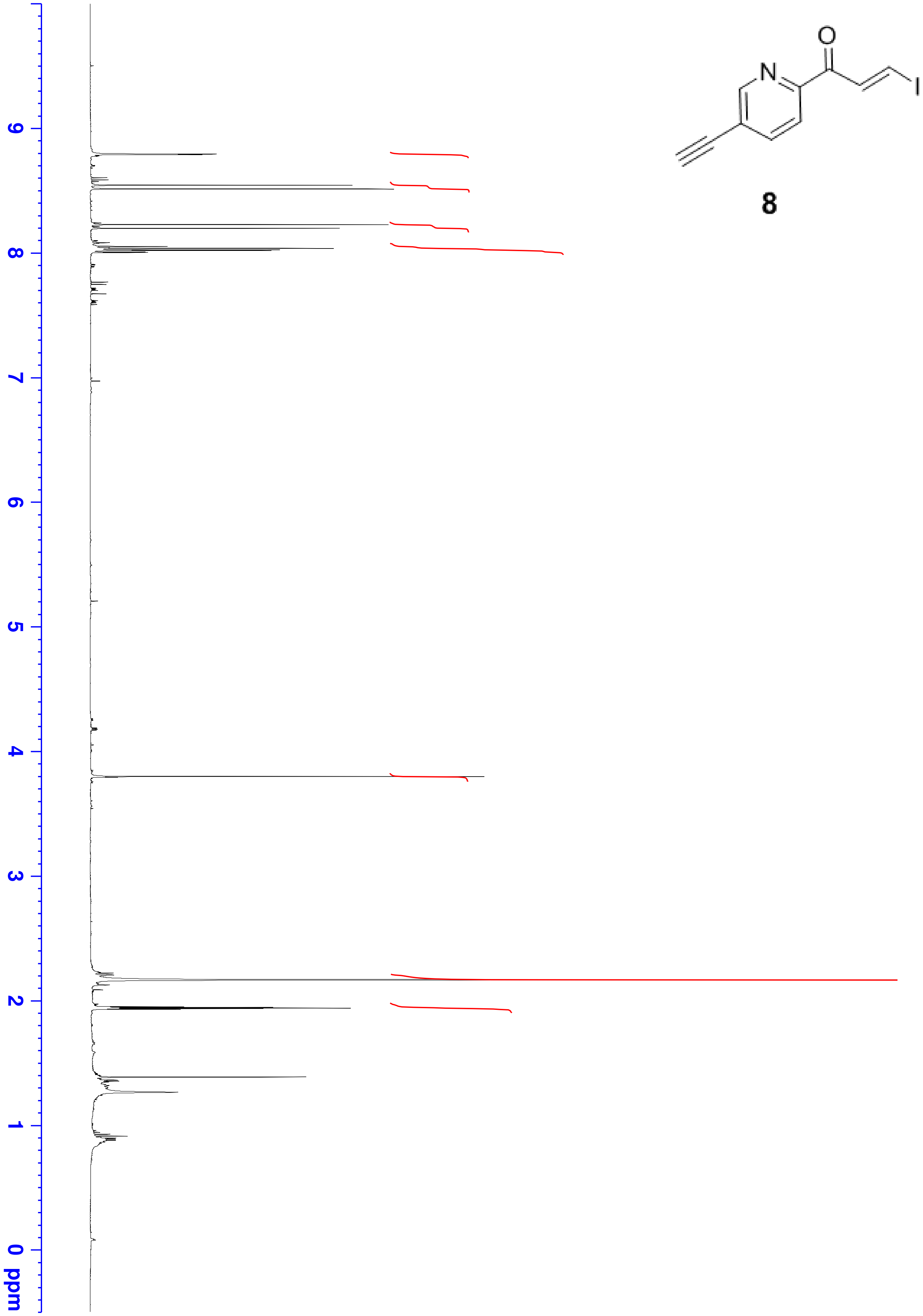

8

^{13}C-NMR DEPT

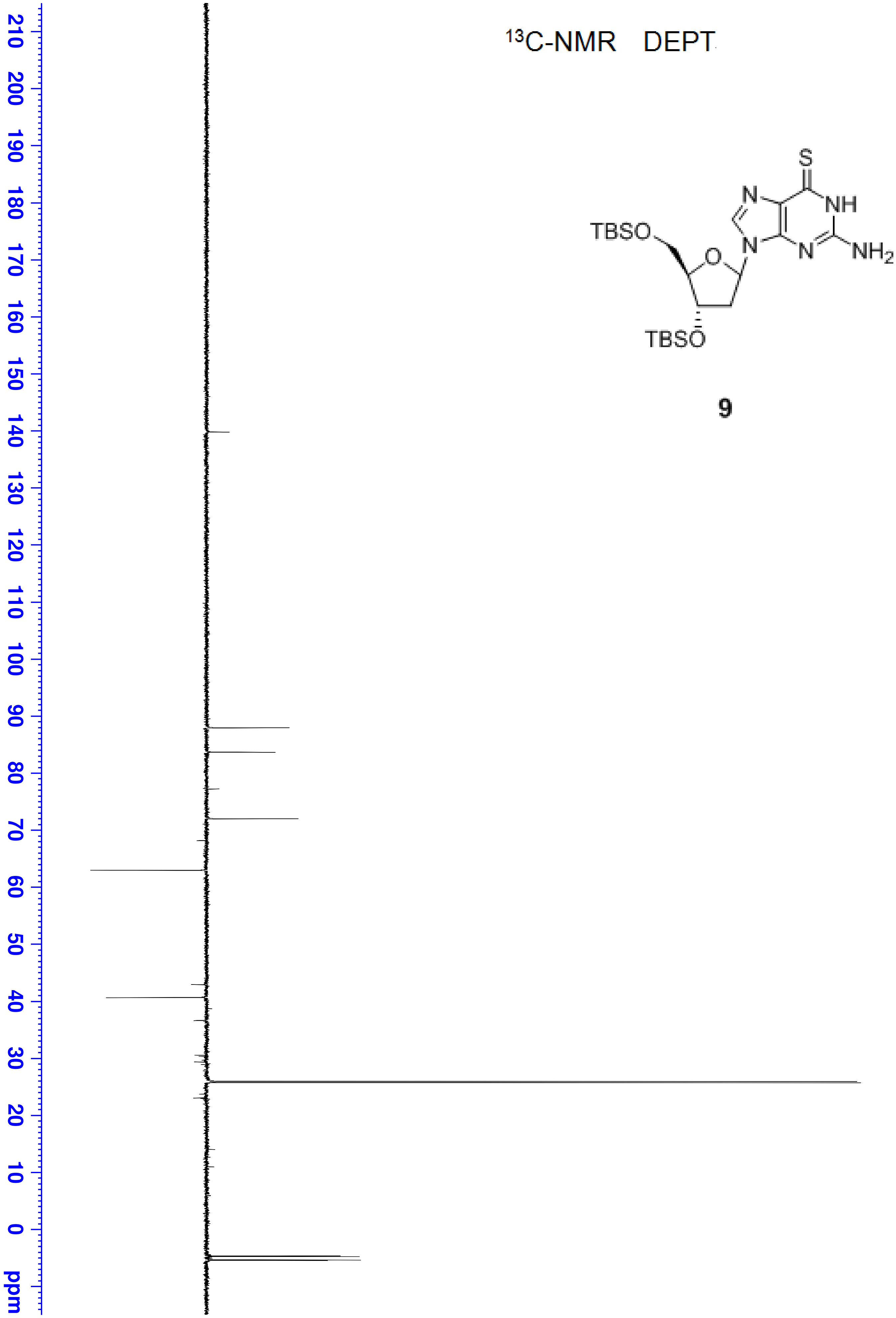

9

^{13}C-NMR

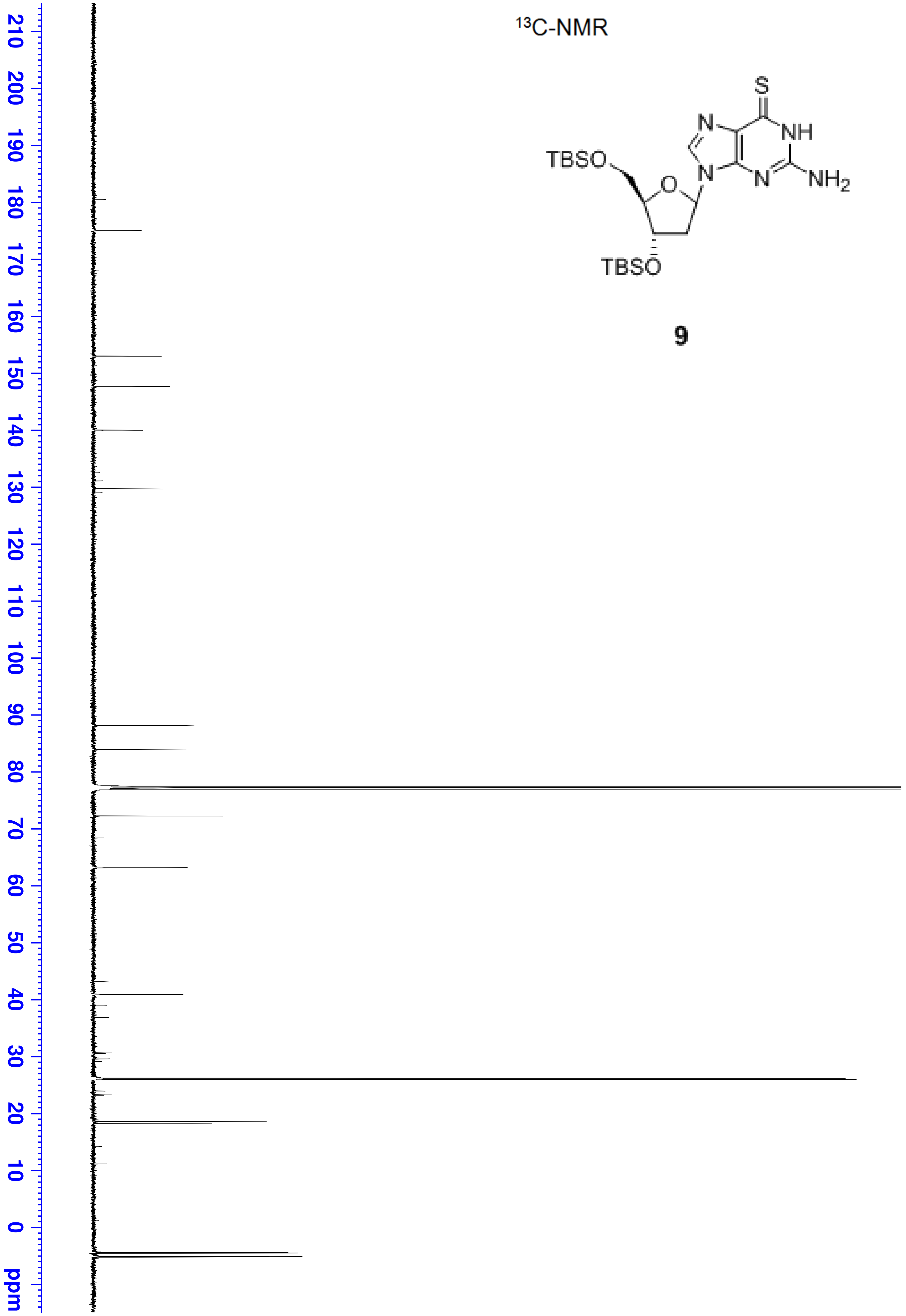

TBSO
TBSO
S
N
NH
N
N
NH2
O
210
200
190
180
170
160
150
140
130
120
110
100
90
80
70
60
50
40
30
20
10
0
ppm

9

dj-559,h1

Pulse Sequence: s2pul
DATE Dec 13 2012
SOLVENT cdcl3
OBSERVE H1
FREQUENCY 399.869 MHz
SPECTRAL WIDTH 10000.0 Hz
ACQUISITION TIME 2.671 sec
RELAXATION DELAY 2.329 sec
PULSE WIDTH 9.0 usec
TEMPERATURE 30.0 deg. C.
NO. REPETITIONS 16
DOUBLE PRECISION ACQUISITION
DATA PROCESSING
RESOLUTION ENHANCEMENT -0.0 Hz
FT SIZE 65536
TOTAL ACQUISITION TIME 1 minutes

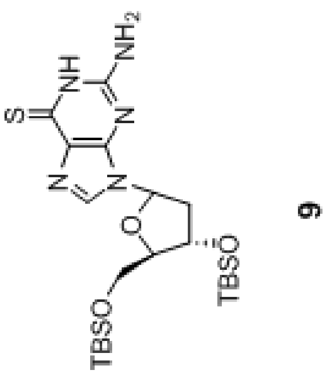

9

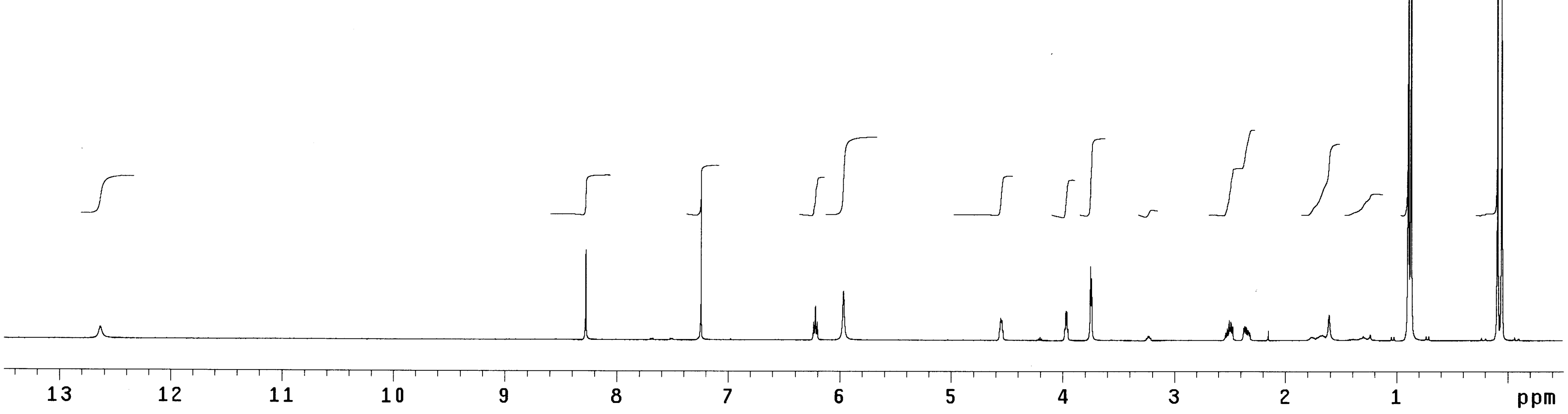

^{13}C-NMR   DEPT

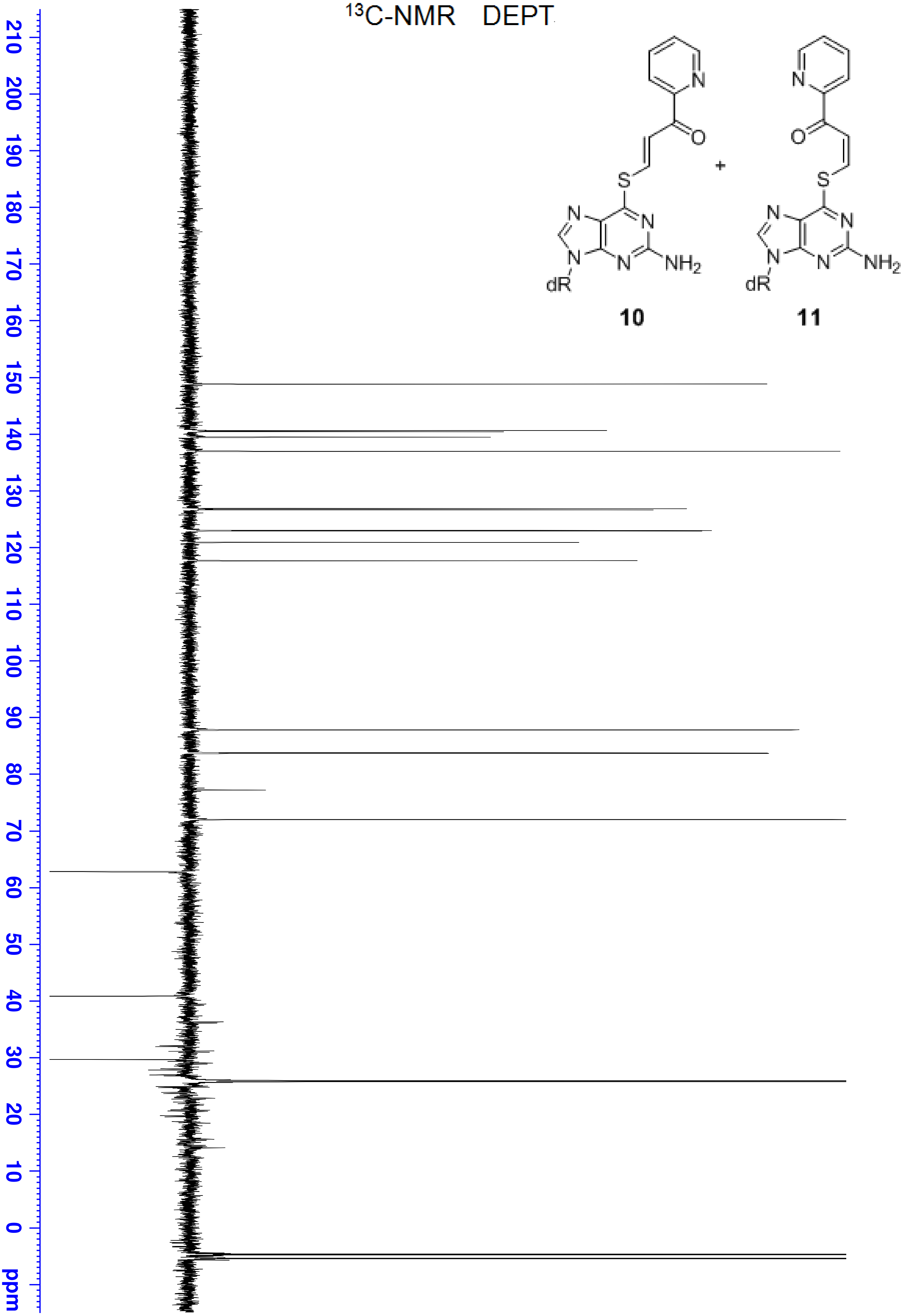
210
200
190
180
170
160
150
140
130
120
110
100
90
80
70
60
50
40
30
20
10
0
ppm
10
11

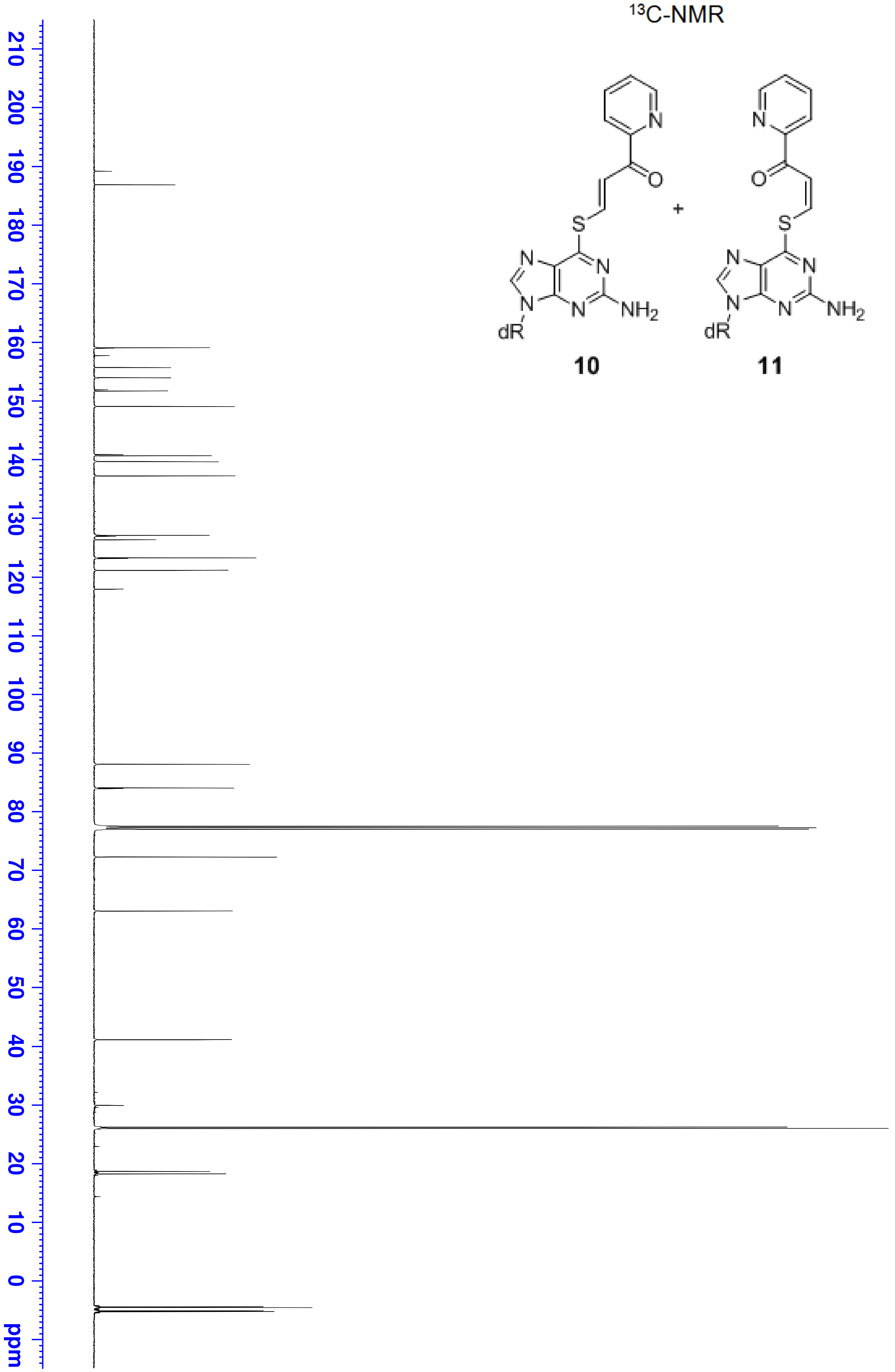

13C-NMR
+
10
11
210
200
190
180
170
160
150
140
130
120
110
100
90
80
70
60
50
40
30
20
10
0
ppm

^{1}H-NMR

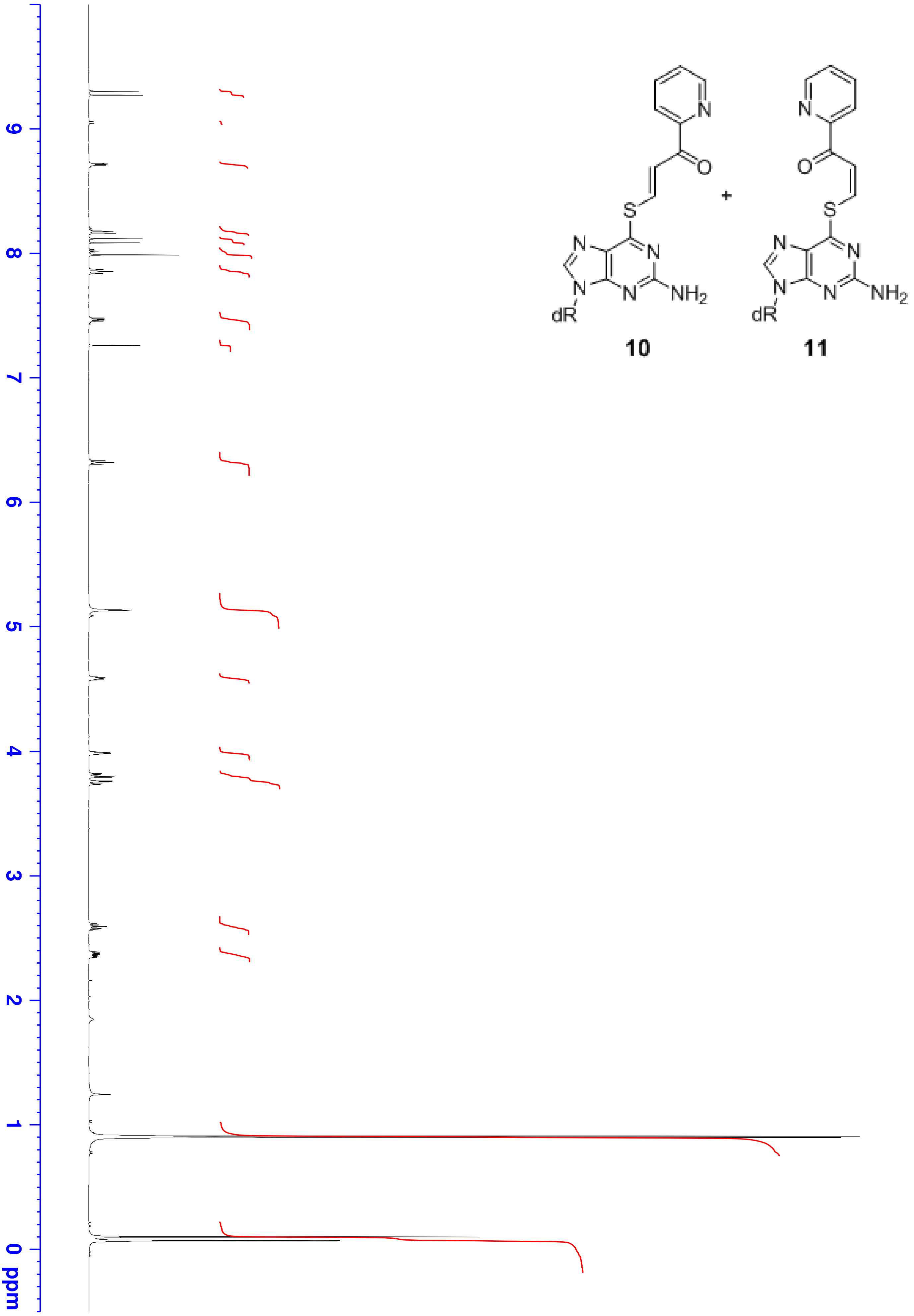

Supplement: SUPPLEMENTARY DATA [file supp_gku538_nar-01309-f-2014-File002.pdf]
